# Aging disrupts the temporal organization of slow oscillations beyond density reduction

**DOI:** 10.1093/pnasnexus/pgag108

**Published:** 2026-04-16

**Authors:** Lucila Capurro, Michael Radloff, María C González, María L Gorosito, Luis I Brusco, Rodrigo Ramele, Cecilia Forcato

**Affiliations:** Laboratorio de Sueño y Memoria, Departamento de Ciencias de la Vida, Instituto Tecnológico de Buenos Aires (ITBA), Iguazú 341, Ciudad Autónoma de Buenos Aires C1437, Argentina; Department of Health Psychology, Institute for Psychology, University of Klagenfurt, Klagenfurt 9020, Austria; Laboratorio de Sueño y Memoria, Departamento de Ciencias de la Vida, Instituto Tecnológico de Buenos Aires (ITBA), Iguazú 341, Ciudad Autónoma de Buenos Aires C1437, Argentina; Laboratorio de Sueño y Memoria, Departamento de Ciencias de la Vida, Instituto Tecnológico de Buenos Aires (ITBA), Iguazú 341, Ciudad Autónoma de Buenos Aires C1437, Argentina; Centro de Neuropsiquiatría y Neurología de la Conducta-CENECON, Facultad de Ciencias Médicas, Universidad de Buenos Aires (UBA), Ciudad Autónoma de Buenos Aires C1121A6B, Argentina; Consejo Nacional de Investigaciones Científicas y Técnicas (CONICET), Ciudad Autónoma de Buenos Aires C1425, Argentina; Departamento de Ingeniería Informática, Instituto Tecnológico de Buenos Aires (ITBA), Ciudad Autónoma de Buenos Aires C1437, Argentina; Laboratorio de Sueño y Memoria, Departamento de Ciencias de la Vida, Instituto Tecnológico de Buenos Aires (ITBA), Iguazú 341, Ciudad Autónoma de Buenos Aires C1437, Argentina

**Keywords:** sleep, aging, electrophysiological changes, slow oscillations

## Abstract

Macroscopic and rhythmic brain oscillations have recently been shown to play a crucial role in glymphatic function by promoting cerebrospinal fluid flow and facilitating the clearance of metabolic waste. While age-related reductions in the number and amplitude of slow oscillations (SOs; 0.5–1 Hz) are well documented and associated with impaired clearance, little is known about how aging affects the temporal structure of these oscillations. Here, we propose that the rhythmic dynamics with which SOs occur represent a critical, yet overlooked, feature supporting glymphatic function. We introduce a novel classification of SOs based on their temporal organization, distinguishing isolated SOs from trains of consecutive oscillations according to inter-SO intervals. Using overnight electroencephalographic recordings from 57 young and 51 elderly adults across three independent datasets, we compared the proportions of isolated and consecutive SOs as well as the distribution of train lengths. Elderly adults displayed a significantly higher proportion of isolated SOs and shorter oscillatory trains than young adults, even after controlling for SO density and stage composition. Temporal shuffling procedures and analyses of density-matched epochs further supported that these differences cannot be attributed solely to density but instead reflect a genuine age-related loss of rhythmicity. These findings reveal that natural aging not only reduces the amount and amplitude of SOs but also disrupts their temporal regularity. This alteration may weaken the sustained ionic currents that drive cerebrospinal fluid flow, compromise the efficiency of metabolic clearance during sleep, and thereby contribute to increased vulnerability to age-related neurodegenerative processes.

Significance statement Sleep slow oscillations (SOs) are critical for multiple brain functions, including memory consolidation and metabolic waste clearance. While previous studies have focused on reductions in SO number and amplitude with aging, our study reveals a novel and overlooked alteration: disruption in their temporal organization, beyond density reduction. By classifying isolated events versus trains of consecutive SOs, we show that elderly adults display fewer sustained SO sequences, indicating a shift toward a less oscillatory brain state. This temporal disorganization may weaken currents that promote cerebrospinal fluid flow, reducing brain clearance efficiency during sleep. Our findings highlight a previously unrecognized mechanism by which normal aging may compromise brain homeostasis and increase risk for neurodegenerative diseases such as Alzheimer's disease.

## Introduction

Slow oscillations (SOs, 0.5–1 Hz) are a hallmark electrophysiological pattern of nonrapid eye movement (NREM) sleep, arising from synchronized cortical neuronal activity (1). In surface electroencephalography (EEG), SOs appear as a phase of deep hyperpolarization followed by a large-amplitude, long-lasting depolarization, typically exceeding 75 µV peak to peak (PTP) in young adults (2). These oscillations are essential for several physiological processes, including synaptic homeostasis (3), memory consolidation (4), immune function (5), and metabolic regulation (6). Notably, NREM sleep and SO power have been shown to enhance cerebrospinal fluid (CSF) flow through the brain parenchyma, facilitating the activity of the glymphatic system responsible for clearing metabolic waste (7–11).

However, aging is associated with a progressive decline in sleep quantity and quality, including greater difficulty falling asleep, more fragmented and lighter sleep, reduced deep sleep, and lower EEG power in both low-frequency bands and fast sleep spindles (12, 13). In particular, SOs decrease not only in total number but also in their density and show reduced amplitude, less steep slopes, and a slight slowing in their average frequency (with a reported ∼0.1 Hz slowing in elderly compared with younger adults) (12, 14, 15). These alterations compromise the physiological processes that rely on them (16–18).

Recently, Jiang-Xie et al. demonstrated that large-scale, rhythmic ionic currents in the brain's interstitial fluid generated by synchronized neuronal activity act as “pumps” that expel metabolic waste from the brain parenchyma (19). Their findings suggest that these high-energy ionic waves, arising from coordinated neuronal activity, are a plausible mechanism underlying the modulation of glymphatic function. Building on this, we propose that cortical SOs (due to their large amplitude, slow periodicity, and coordinated cortical propagation) may be key drivers of these ionic currents, thus playing an active role in supporting glymphatic function during NREM sleep.

While previous studies have associated the age-related decline in glymphatic activity primarily with reductions in SO power (8), we propose that the temporal distribution of SOs may also be a critical yet overlooked feature. Given that CSF follows a directional pathway for waste removal from the brain parenchyma (20), we reason that if SOs contribute to CSF movement (as has been suggested), then not only their amplitude and density but also their temporal spacing may be critical. Sustained and rhythmically organized oscillations could generate more continuous ionic currents to support this flow, whereas the same number of oscillations dispersed over longer intervals may be less effective. Accordingly, we hypothesize that aging may impair not only the generation of large-amplitude SOs but also their temporal organization, thereby disrupting sustained currents, reducing glymphatic clearance, and promoting the accumulation of toxic proteins such as beta-amyloid implicated in Alzheimer's disease (21–25). Although we do not directly measure CSF flow in the present study, this hypothesis motivated us to investigate, for the first time, the temporal dynamics of SOs in younger and older adults.

Here, we introduce a novel classification that distinguishes SOs based on their temporal spacing: isolated SOs, which occur individually and are temporally distant from others, and consecutive SOs, which appear in sequence to form SO trains of varying length. The length of a train is defined by the number of consecutive SOs it comprises, with longer trains reflecting a more sustained rhythmic structure. Using overnight EEG recordings from young and elderly adults across three independent datasets, we quantified the proportion of isolated versus consecutive SOs and characterized the distribution of train lengths.

Elderly adults exhibited a higher proportion of isolated SOs and shorter SO trains compared with young adults. These findings suggest that aging not only reduces the number and amplitude of SOs but also disrupts their temporal organization, shifting brain activity toward a less rhythmic state that may compromise glymphatic clearance during sleep.

## Results

### Age-related differences in the temporal organization of SOs

**Figure 1:**
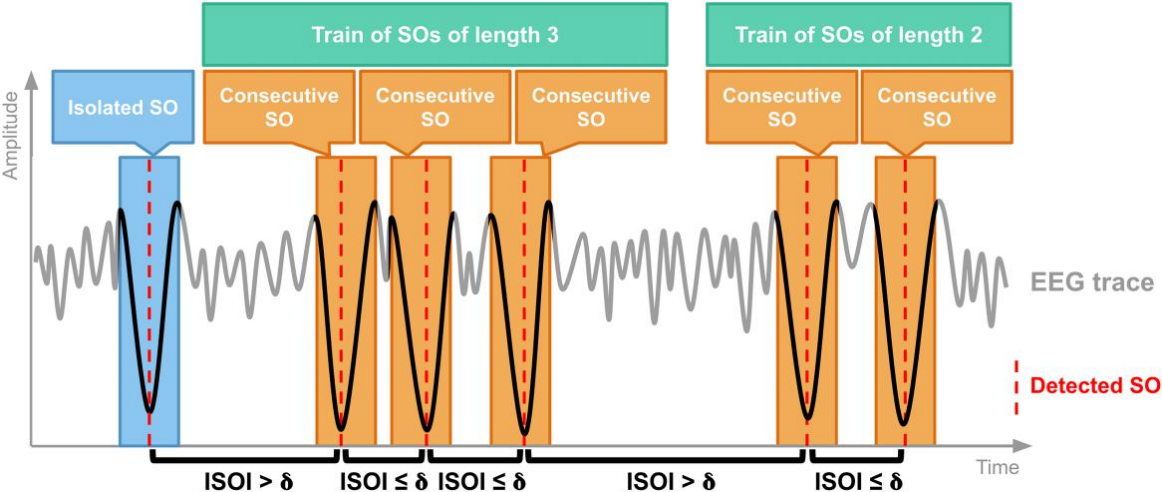
Schematic illustration of SO classification. The trace shows an example EEG segment in which detected SOs (red dashed lines) are categorized according to their temporal separation, quantified using the ISOIs. An isolated SO (blue) is defined as a wave occurring >*δ* seconds apart from any other SO, whereas a consecutive SO (orange) occurs within *δ* seconds of another SO. Trains of SOs (green) represent sequences of consecutive SOs, with train length defined as the number of waves contained in each sequence. SO, slow oscillation; ISOI, inter-SO interval.

We analyzed polysomnographic recordings from whole nights of sleep of 57 young and 51 elderly adults, pooled from three independent datasets (see Materials and methods for details on datasets and preprocessing). To ensure that our conclusions were not driven by dataset-specific factors, we first conducted all analyses independently within each dataset, and upon observing consistent results across datasets, we proceeded to perform pooled analyses combining all subjects. SOs were detected in artifact-free NREM (stage 2 and slow-wave sleep [SWS]) epochs and classified as isolated or trains of consecutive SOs based on inter-SO intervals (ISOIs) and the definition of a threshold based on the upper limit of the SO period (*δ* = 2 s; Fig. 1; see Materials and methods). We compared the proportions of isolated and consecutive SOs, as well as SO train lengths, between age groups. These analyses were conducted altogether in NREM sleep, but stage-specific analyses were also performed separately in stage 2 and SWS, and further analyses were done to account for differences in SO density between groups (14).

#### Elderly adults showed a higher proportion of isolated SOs and shorter SO trains

**Figure 2:**
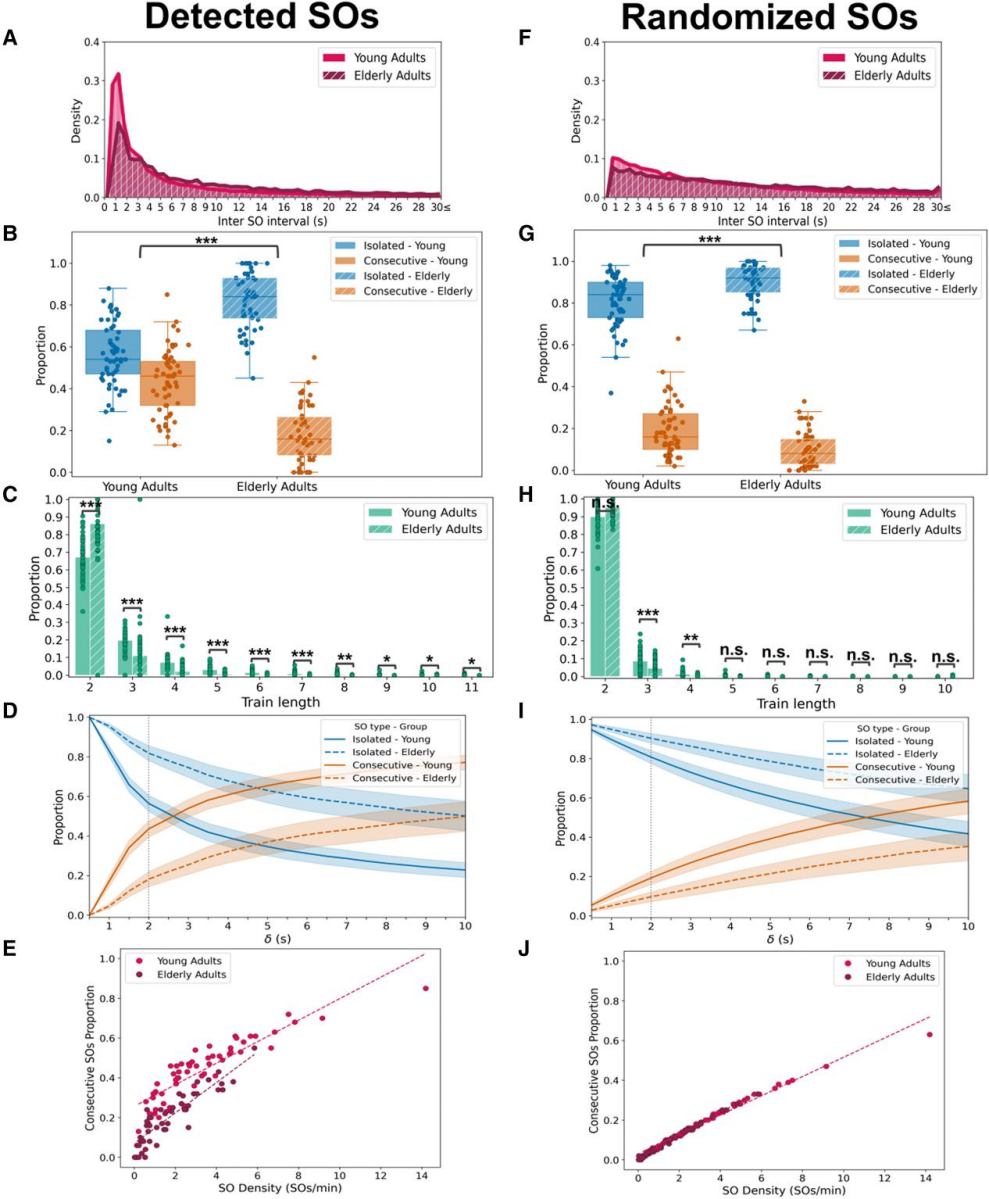
Characterization of SO types and train structure during NREM sleep in the pooled dataset. Results are shown for detected (left panels, A–E) and temporally randomized (right panels, F–J) SOs; randomization preserved SO density per subject while shuffling SO time points within artifact-free NREM epochs. Data are shown for young (*n* = 57) and elderly (*n* = 51) adults. A, F) ISOI density histograms showing the distribution of time (s) between successive SO negative peaks in young (solid magenta) and elderly (striped burgundy) adults. B, G) Proportions of isolated (blue) and consecutive (orange) SOs across age groups. Boxplots show distributions for young (solid boxes) and elderly (striped boxes) adults. The statistical comparison tests whether the two-type composition of SOs differs between age groups. C, H) Train length distributions. Density histograms indicate the relative frequency of trains of lengths 2 to 11 for young (solid bars) and elderly (striped bars) adults. D, I) SO type proportions as a function of the ISOI threshold (*δ*). Mean proportions of isolated (blue) and consecutive (orange) SOs are shown for *δ* ranging from 0.5 to 10 s. Solid lines indicate young adults; dashed lines indicate elderly adults. Shaded areas represent 95% CIs. The vertical dotted line marks the 2-s threshold used in the main analysis. E, J) Relationship between global SO density (waves/minute of NREM sleep) and the proportion of consecutive SOs per subject. Linear fits are shown for young (magenta) and elderly (burgundy) adults. ***P < 0.001; **P < 0.01; *P < 0.05.

ISOI distributions (Fig. 2A) showed significant differences in the temporal structure of SOs between age groups (two-sample Kolmogorov–Smirnov test: *D* = 0.218, *P* < 0.001). Both distributions had their peak at ISOI values <2 s (in both groups, the bin with the highest event count corresponded to ISOIs between 1 and 1.5 s), supporting the decision of using *δ* = 2 s. However, the distribution for young adults was more sharply peaked and declined more steeply (peak density = 0.317), whereas that of elderly adults was flatter with a lower peak (0.191) and a broader spread toward longer ISOIs, indicating that young adults exhibited a greater tendency for SOs to cluster in trains while elderly adults showed more temporally dispersed SOs with fewer trains. This broader dispersion in the elderly group was further supported by the log-normal fit, which showed a significantly larger shape parameter (*σ* elderly = 1.544 vs. *σ* young = 1.449; Δ*σ* = −0.095, 95% CI [−0.116, −0.075], *P* < 0.001), consistent with a heavier-tailed distribution.

When analyzing the proportion of isolated and consecutive SOs, we found that the elderly adults exhibited a higher proportion of isolated SOs and a reduced proportion of consecutive SOs compared with younger adults (Fig. 2B; mean ± SEM isolated proportion: young = 0.56 ± 0.02, elderly = 0.82 ± 0.02; beta regression with random intercepts: *β*1 = 0.94, *z* = 6.58, *P* < 0.001). Additionally, we found that train lengths were significantly shorter for elderly adults (Fig. 2C). Specifically, elderly adults exhibited a higher proportion of the shortest possible trains (length = 2; *P* < 0.001) and a lower proportion of trains with lengths ranging from 3 to 11 (all *P*s < 0.05). Full descriptive statistics (mean ± SEM) and complete Dirichlet regression results for each train length are provided in Table S1.

To ensure that these findings were not dependent on the specific threshold chosen for classifying SOs, we varied *δ* and measured the resulting proportions of isolated and consecutive SOs (Fig. 2D). As expected, when *δ* < 1 s, all SOs were classified as isolated. With increasing *δ*, the proportion of isolated SOs decreased while consecutive SOs increased. This shift was markedly steeper in young adults, who consistently showed more consecutive SOs across the full range of thresholds. This result indicates that age-related differences are robust across thresholds (note that values much larger than 2 s no longer represent oscillatory trains; the analysis serves only to support that the observed group differences and conclusions are not dependent on the exact choice of a 2-s threshold).

To test whether the age-related differences in SO dynamics could simply reflect disparities in SO density, we first analyzed the proportion of consecutive SOs as a function of global SO density across NREM sleep in both groups (Fig. 2E). As expected, a positive association between SO density and consecutive SO proportion was observed in both groups, indicating that higher SO density is associated with a higher proportion of SOs forming trains. Multiple regression analysis with mean-centered SO density revealed a significant interaction between group and SO density, indicating that the relationship between density and the proportion of consecutive SOs differs between younger and elderly adults (density × group = −0.030, *t*(104) = −4.25, *P* < 0.001; density = 0.084, *t*(104) = 13.56, *P* < 0.001; group = 0.134, *t*(104) = 9.758, *P* < 0.001; *R*^2^ = 0.89). The significant group effect indicates that younger adults with an average SO density (2.51) had a higher predicted proportion of consecutive SOs when compared with elderly adults with the same SO density.

#### SO density alone cannot account for the observed group differences

To further investigate whether these differences could be explained by the reduction in NREM SO density with aging (14) or whether they reflect an age-related disruption in the temporal organization of SOs, we performed a temporal shuffling procedure (Fig. 2, right column panels). SOs were redistributed randomly within NREM epochs, preserving overall density while disrupting their temporal structure (see Materials and methods section). In this approach, the empirical data are referred to as the observed dataset, while the shuffled version constitutes the randomized dataset. If the group differences in SO type proportions remained unchanged after this procedure, it would suggest that density alone completely accounts for the observed effects. Conversely, a change in the results after randomization would indicate that the temporal arrangement of SOs plays a role beyond mere density.

This comparison revealed clear differences between observed and randomized datasets (Fig. 2A and F). A significant Kolmogorov–Smirnov test indicates differences between real and randomized distributions (young: *D* = 0.284, *P* < 0.001; elderly: *D* = 0.181, *P* < 0.001). In the observed data, both groups showed a prominent peak at short ISOIs (<2 s), with the highest-density bin centered at 1–1.5 s, indicating a strong tendency for SOs to cluster into trains (Fig. 2A). In contrast, in the randomized dataset, this peak disappeared in both groups, and the distributions became flatter, indicating that SO trains are lost when temporal structure is disrupted (Fig. 2F). The resulting distribution was flatter in elderly adults due to the lower SO density (peak density young = 0.102 vs. elderly = 0.077), allowing shuffled SOs to spread more evenly across NREM epochs. In contrast, despite the randomization, young adults retained a mild skew toward shorter intervals, as the high SO density in this group increases the probability of shorter intervals even after randomization (log-normal fit showed a significantly larger shape parameter: *σ* elderly = 1.544 vs. *σ* young = 1.449; Δ*σ* = −0.095, 95% CI [−0.116, −0.075], *P* < 0.001).

Randomization significantly altered group differences in the proportion of isolated SOs (Fig. 2B and G; mean ± SEM isolated proportion: young = 0.81 ± 0.02, elderly = 0.90 ± 0.01; beta regression with the “elderly” group and “observed” dataset as reference categories: *β*3 = −0.56, *z* = −2.92, *P* = 0.004, where *β*3 reflects the group × dataset interaction) and in train lengths between 2 and 8 (all *P*s < 0.05; Fig. 2C and H). This suggests that the formation of longer SO trains in young adults is not merely a consequence of increased density but reflects a preserved temporal structure that is disrupted in aging. Full descriptive statistics (mean ± SEM) and complete Dirichlet regression results for each train length are reported in Table S2. Even though the group difference in the randomized dataset reaches significance when analyzed individually (Fig. 2G; mean ± SEM isolated proportion: young = 0.81 ± 0.02, elderly = 0.90 ± 0.01; beta regression with random intercepts: *β*1 = 0.67, *z* = 4.49, *P* < 0.001), with additional significant effects for trains of length 2 and 3 (*P* < 0.05; Fig. 2H; Table S3), the interaction term in the model including dataset (observed vs. randomized) bolsters the assumption that the distributions of SO proportions differ beyond chance level between the observed and randomized datasets.

Sensitivity analyses of the *δ* parameter further supported this conclusion. In the observed dataset, the proportion of isolated versus consecutive SOs changed sharply with *δ*, with steeper slopes in young adults and a clear separation between groups (Fig. 2D). In the randomized dataset, this transition became flatter in both groups, reflecting a general loss of oscillatory structure, particularly in the younger group (Fig. 2I).

Moreover, when examining the relationship between global SO density and the proportion of consecutive SOs, we tested whether group differences varied across the observed and randomized datasets (Fig. 2E and J). Visual inspection suggested that differences apparent at comparable density levels in the observed dataset were lost after randomization and that the regression line for SO density was flatter for elderly adults in the shuffled data when compared with the observed data. The significant three-way interaction between group, density, and dataset is in line with that observation (multiple linear regression with mean-centered density: density = 0.08, *t*(208) = 18.66, *P* < 0.001; group = 0.14, *t*(208) = 13.439, *P* < 0.001; dataset = −0.11, *t*(208) = −9.754, *P* < 0.001; density × group = −0.03, *t*(208) = −5.84, *P* < 0.001; density × dataset = −0.026, *t*(208) = −4.101, *P* < 0.001; group × dataset = −0.14, *t*(208) = −9.61, *P* < 0.001; density × group × dataset = 0.02, *t*(208) = 2.89, *P* = 0.004; *R*^2^ = 0.93). To further explore the three-way interaction, we analyzed the shuffled data separately the same way we analyzed the observed data. We found a significant interaction between group and density. In addition, and in sharp contrast to the pronounced group difference at average SO density in the observed data, the group difference between simulated elderly and simulated young participants at average SO density levels was estimated close to zero and nonsignificant (multiple linear regression with mean-centered density: density = 0.13, *t*(104) = 39.42, *P* < 0.001; group = −0.00, *t*(104) = −0.432, *P* = 0.67; density × group = −0.02, *t*(104) = −5.34, *P* < 0.001; *R*^2^ = 0.98). These results suggest that NREM SO density alone is not a plausible explanation for the observed group differences, which we conclude depend on the preservation of temporal structure.

#### Elderly adults showed altered SO rhythmicity in both stage 2 and SWS

We then investigated whether the higher proportion of isolated SOs in elderly adults could be explained by the relative composition of stage 2 and SWS within NREM, given that elderly adults typically have reduced SWS and increased stage 2 (12), and these stages differ in their intrinsic SO density. To disentangle stage composition effects, we analyzed SO temporal organization separately in stage 2 and SWS. In both stages, elderly adults consistently showed a higher proportion of isolated SOs and shorter trains compared with younger adults. These effects remained significant after controlling for SO density and across a range of thresholds (see Fig. S2 and Tables S4 and S5 for full results).

#### Elderly adults showed a higher proportion of isolated SOs even in density-matched epochs

**Figure 3:**
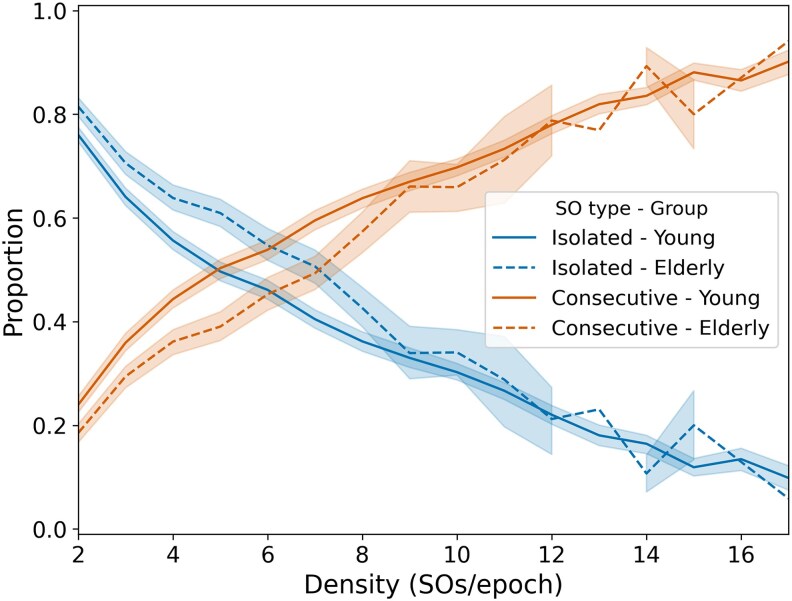
Relationship between SO type proportions and local SO density during NREM sleep in the pooled dataset. For each 30-s NREM epoch, the proportion of isolated (blue) and consecutive (orange) SOs was computed and then averaged across epochs with the same density (number of SOs per epoch). Data are shown for young (*n* = 57) and elderly (*n* = 51) adults. Solid lines correspond to young adults and dashed lines to elderly adults. Shaded areas represent 95% CIs; CIs are omitted for density bins with insufficient data.

Finally, to directly control for density effects (14), we conducted an epoch-based analysis by comparing the proportions of isolated and consecutive SOs within density-matched epochs between groups (see Materials and methods). Naturally, epochs with very low SO counts (e.g. 2 SOs) tend to consist of isolated SOs, while epochs with very high counts (e.g. ≥10 SOs) are more likely to contain consecutive SOs due to space constraints. The most informative range lies in epochs with intermediate densities, where the same number of SOs could either form trains or remain isolated depending on their temporal organization. While both groups showed an increase in the proportion of consecutive SOs with density, younger adults consistently exhibit higher proportions of trains across intermediate densities (4–9 SOs/epoch; Fig. 3). Therefore, even under this strict control, elderly adults exhibited more isolated SOs, further supporting the finding that age-related changes in SO temporal organization are not simply a consequence of density differences.

#### Findings are consistent across three independent datasets

**Figure 4:**
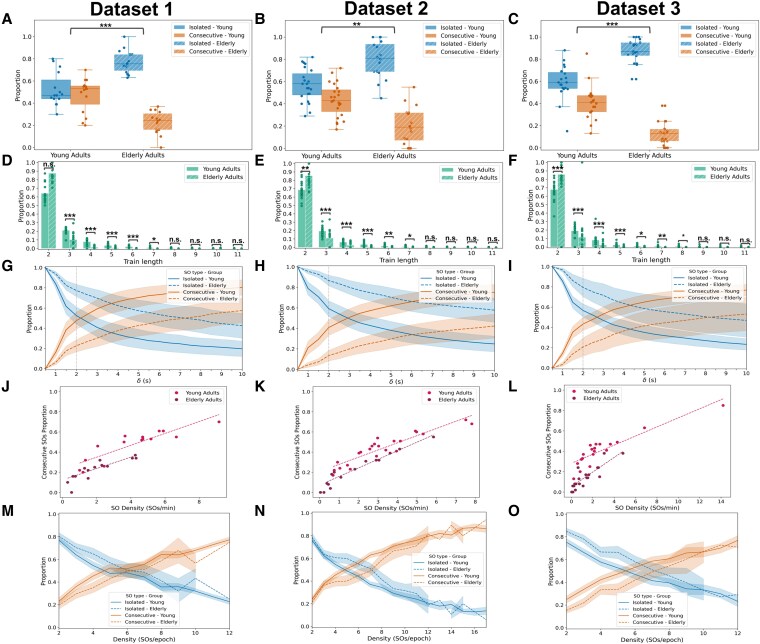
Replication of SO type and train length analyses during NREM sleep across individual datasets. Data are shown separately for three independent datasets: dataset 1 (young *n* = 15, elderly *n* = 14), dataset 2 (young *n* = 23, elderly *n* = 16), and dataset 3 (young *n* = 19, elderly *n* = 21). A–C) Proportions of isolated (blue) and consecutive (orange) SOs across age groups. Boxplots show distributions for young (solid boxes) and elderly (striped boxes) adults. The statistical comparison tests whether the two-type composition of SOs differs between age groups. D–F) Train length distributions. Density histograms indicate the relative frequency of trains of lengths 2 to 11, for young (solid bars) and elderly (striped bars) adults. G–I) SO type proportions as a function of the ISOI threshold (*δ*). Mean proportions of isolated (blue) and consecutive (orange) SOs are shown for *δ* ranging from 0.5 to 10 s. Solid lines indicate young adults; dashed lines indicate elderly adults. Shaded areas represent 95% CIs. The vertical dotted line marks the 2-s threshold used in the main analysis. J–L) Relationship between global SO density (waves/minute of NREM sleep) and the proportion of consecutive SOs per subject. Linear fits are shown for young (magenta) and elderly (burgundy) adults. M–O) For each 30-s NREM epoch, the proportion of isolated (blue) and consecutive (orange) SOs was computed and then averaged across epochs with the same density (number of SOs per epoch). Solid lines correspond to young adults, dashed lines to elderly adults. Shaded areas represent 95% CIs; CIs are omitted for density bins with insufficient data. ***P < 0.001; **P < 0.01; *P < 0.05.

While the pooled dataset provided the primary results presented above, our analyses were first conducted independently within each dataset. Despite differences in recording protocols, elderly adults consistently exhibited more isolated SOs and shorter trains across datasets, with results robust to threshold variations and density matching (see Fig. 4 and Tables S6–S8 for full dataset-specific analyses). Minor variations did not alter the overall pattern, supporting the robustness and generalizability of the findings.

Together, these analyses indicate that the observed group differences are not reducible to global density differences, stage composition, density fluctuations across epochs, or dataset-dependent factors, but rather reflect robust age-related alterations in the temporal organization of SOs.

#### Standard sleep analyses replicate known age-related changes

Additionally, we conducted standard analyses of sleep architecture, power spectral density (PSD), and SO features to validate that our dataset captures well-documented age-related changes in sleep physiology (12–15) and to obtain a comprehensive overview of sleep patterns across groups.

Elderly adults showed greater sleep fragmentation, reduced rapid eye movement (REM) and NREM sleep (particularly slow-wave sleep), and a redistribution toward lighter sleep. Low-frequency activity (SO and delta bands) and fast spindle power were reduced, and SOs were fewer, smaller in amplitude, slower, and shallower, consistent with prior literature (12–15). Detailed results are provided in Tables S9–S12.

Given the marked increase in sleep fragmentation in the elderly group (more awakenings and higher sleep fragmentation index [SFI]; Table S9), we next examined whether sleep fragmentation could account for age-related differences in the proportion of isolated SOs. Visual inspection of the data revealed largely parallel relationships between fragmentation measures and isolated SO proportion in both groups, with elderly adults consistently exhibiting higher isolated SO proportions across the full range of fragmentation values (Fig. S3). Consistent with this observation, multiple linear regression models revealed a significant group effect but no significant effect of fragmentation nor a group × fragmentation interaction. This pattern held when fragmentation was indexed either by the number of awakenings or by the SFI, indicating that increased sleep fragmentation alone does not explain the higher prevalence of isolated SOs observed in older adults (awakenings model: group = −0.273, *t*(104) = −4.43, *P* < 0.001; awakenings = 0.001, *t*(104) = 0.20, *P* = 0.841; awakenings × group = 0.002, *t*(104) = 0.41, *P* = 0.683; *R*^2^ = 0.45; SFI model: group = −0.272, *t*(104) = −4.13, *P* < 0.001; SFI = 0.010, *t*(104) = 0.82, *P* = 0.413; SFI × group = 0.009, *t*(104) = 0.51, *P* = 0.611; *R*^2^ = 0.46).

## Discussion

We propose a new method to analyze changes in SO dynamics as a consequence of the natural aging process. While previous research has predominantly focused on the quantity and amplitude of SOs (12, 14, 15), we incorporate a new approach to study alterations in the rhythmicity and periodicity of these oscillations.

To the best of our knowledge, we found for the first time that elderly adults exhibit a higher proportion of isolated SOs and shorter trains of consecutive SOs compared with those observed in young adults. These findings lead us to propose that natural aging shifts the brain toward a less oscillatory state, characterized by a diminished ability to produce sustained, periodic oscillations.

Our findings extend current understanding of sleep-related brain aging by revealing a loss of temporal rhythmicity in SOs that goes beyond previously described reductions in density and amplitude. Through SO randomization analysis and density-matched controls, we demonstrate that age-related differences in the temporal distribution of SOs cannot be explained by a reduction in density alone (14) but instead reflect a loss of rhythmic temporal structure.

These age-related differences were evident in both stage 2 and SWS, supporting the idea that the observed age-related differences in SO dynamics cannot be solely attributed to a global reduction in SWS and a relative increase in stage 2 sleep in elderly adults (where different oscillatory dynamics are expected by definition) (12) but rather reflect alterations in the temporal organization of SOs that persist within each NREM stage.

Importantly, these results were replicated across three independent datasets that differed in acquisition systems, scoring criteria, and recording environments. The consistency of the findings across these heterogeneous datasets highlights the robustness and generalizability of the observed age-related alterations in SO dynamics.

This disruption in oscillatory rhythmicity may have functional implications. Previous research has established that effective clearance of metabolic waste requires continuous CSF flow, driven by periodic arterial pulsations and improved by the increased interstitial space during SWS (7–11). However, recent studies have shown that large-scale, rhythmic ionic currents in the brain's interstitial fluid generated by synchronized neuronal activity act as “pumps” that expel metabolic waste from the brain parenchyma (19). Their findings suggest that these high-energy ionic waves, arising from coordinated neuronal activity, are a plausible mechanism underlying the modulation of glymphatic function. Building on this, we propose that cortical SOs (due to their large amplitude, slow periodicity, and coordinated cortical propagation) may be key drivers of these ionic currents, thus playing an active role in supporting glymphatic function during NREM sleep.

Integrating these findings and our results, we propose that not only the amount and amplitude of SOs but also their temporal rhythmicity serves a critical function in brain homeostasis. While the age-related decline in glymphatic activity has been linked to reduced SO power (8), we propose that this deterioration is not solely due to reductions in amplitude and density but also to disruptions in the temporal organization of SOs. Given that CSF follows a directional pathway for waste removal from the brain parenchyma (20), we argue that sustained ionic currents are essential for effective glymphatic function. Accordingly, we hypothesize that aging compromises not only the generation of large-amplitude SOs but also impairs the brain's ability to sustain temporally structured trains of SOs, potentially disrupting CSF dynamics, reducing the efficiency of metabolic waste clearance during sleep, and contributing to age-related neurodegenerative processes (21–25).

Future research should explore the link between consecutive SO rhythmicity and glymphatic function. Animal models, where rhythmicity can be experimentally modulated while keeping the number of consecutive SOs constant, could help elucidate the relationship between the oscillatory capacity and brain clearance.

Although in the present work we discuss the implications of disrupted SO rhythmicity for glymphatic function given its direct dependence on sustained periodic neural activity (12), it is important to note that other sleep-dependent processes traditionally linked to SO density and slow-wave continuity may also be influenced by this previously uncharacterized aspect of SO dynamics. Recent studies have begun to distinguish multiple morphological types of slow waves (26–31), highlighting an increasing appreciation of the heterogeneity of SOs. In this context, the temporal classification introduced here adds an additional and complementary dimension of complexity to a more comprehensive characterization of SOs. Importantly, SO functionality may not be determined solely by morphology or waveform subtype but also by temporal organization and rhythmic structure (for a related analysis of waveform features across temporal categories, see Supplementary Results). This perspective opens new avenues for investigating the specific contribution of SO rhythmicity to broader aspects of sleep physiology, such as synaptic homeostasis, learning and memory consolidation (3) or emotional regulation (32, 33).

Finally, the proposed classification of SOs into isolated events and trains enables the calculation of their respective proportions and train lengths. These derived metrics offer the opportunity to establish new biomarkers of neurophysiological aging and to guide the development of therapeutic strategies aimed at preserving or restoring the brain's ability to sustain temporally organized SO activity.

### Limitations

One limitation of this study lies in the methodological heterogeneity across individual datasets, including differences in EEG acquisition systems (clinically validated vs. wearable devices), number and placement of channels, referencing schemes, sleep scoring criteria (American Academy of Sleep Medicine [AASM] vs. Rechtschaffen and Kales [R&K]), and recording environments (laboratory vs. home settings). This heterogeneity could introduce bias when pooling the datasets. However, it does not affect intradataset comparisons, as the young and elderly groups within each dataset (except for dataset 1) were retrieved from the same study. In the case of dataset 1, different EEG acquisition systems were used for each group: young adults were recorded with a BrainAmp system and elderly adults with OpenBCI devices. Nonetheless, previous studies have validated the OpenBCI device against medical-grade devices like Brain Products (34–37) and have shown that it is a suitable and reliable tool for sleep EEG research. However, this variability also represents a strength, as consistent age-related patterns were observed across independent datasets supporting the robustness and generalizability of our findings.

Additionally, a limitation of the current approach is that the interevent interval threshold (*δ*) used to classify SOs as isolated events or part of oscillatory trains was fixed at 2 s. Although this threshold was chosen based on the upper limit of the SO period and supported by sensitivity analyses, it does not account for variations in SO characteristics, such as waveform duration and frequency. Given that the actual period of SOs typically ranges between ∼1 and 2 s (corresponding to 0.5–1 Hz), using a fixed threshold may fail to capture rhythmicity accurately across all SOs. Future work could benefit from the use of adaptive or waveform-informed thresholds tailored to individual oscillatory properties to more precisely characterize the temporal structure of SO activity.

A further limitation concerns the detection of SOs itself. There is currently no universally accepted standard algorithm for automatic SO detection (38), and even studies that rely on “fixed” amplitude thresholds do not use the same values (2, 14, 39), leading to substantial variability across the literature. Other approaches apply age- or sex-adjusted thresholds (15, 40) or adaptive methods (41–43), all of which can influence the number, morphology, and timing of detected events. A systematic evaluation of how these different strategies affect SO detection is beyond the scope of the present work and would require a dedicated methodological study. Future investigations should aim to refine detection criteria to better account for age-related changes in SO morphology and additionally incorporate sex-specific thresholds that enable a more precise characterization of potential differences between men and women. Establishing such standardized and biologically informed criteria would make it possible to examine how SO rhythmicity evolves across the lifespan and whether alterations follow distinct trajectories in men and women.

Additionally, several demographic and clinical variables that may modulate SO characteristics such as body mass, sleep quality, physical activity, medication use, or other health-related factors were not examined here due to their inconsistent availability across datasets. Incorporating richer demographic and clinical metadata in future work will be important for better understanding their contribution to age-related changes in SO activity.

## Materials and methods

### Subjects

We analyzed overnight polysomnography (PSG) recordings from whole nights of sleep of a total of 108 subjects, divided into 57 young adults (aged 20–35 years, mean = 25.8 ± 0.4 years; 35 women, 22 men) and 51 elderly adults (aged 60–85 years, mean = 68.4 ± 4.3 years; 32 women, 19 men). These age ranges were selected based on common definitions of “young” (∼20–30 years) and “older” (∼60–80 years) adults used in cognitive neuroscience research (38, 44, 45). These data were pooled from three independent datasets, each collected in different studies with its own methodological particularities, including differences in acquisition systems, environments, and study protocols. By combining multiple datasets, we aimed to increase the robustness of our findings and assess their generalizability across different recording conditions. To assess the generalizability of our findings and account for potential limitations associated with relying on a single dataset, we conducted both pooled analyses across all datasets and separate analyses within each individual dataset. Details for each dataset are described below. A full list of subject codes included from each dataset is provided in Table S15.

#### Dataset 1

This dataset included recordings from a total of 29 subjects: 15 young (aged 20–30 years, mean = 24.2 ± 0.7 years; 12 women, 3 men) and 14 elderly (aged 60–85 years, mean = 68.4 ± 4.8 years; 12 women, 2 men) adults. Young adults' data were sourced from a publicly available dataset from the previous study by Forcato et al. (46). All 15 subjects from the control group “NR” of experiment 3, which involved full nights of sleep, were included. No subjects from this group were excluded from the analysis. Elderly adults' data were sourced from a dataset from the Laboratorio de Sueño y Memoria at the Instituto Tecnológico de Buenos Aires as part of a study investigating acoustic stimulation of SOs in elderly adults. All control subjects who did not receive any acoustic stimulation were included in the present study. Participants were recruited through the lab's social media platforms and were screened to exclude severe medical, neurological, or psychiatric conditions. The study protocol was reviewed and approved by the Comité de Ética Humana, Facultad de Ciencias Médicas, Universidad de Buenos Aires. All participants provided written informed consent prior to participation. Subjects from both age groups underwent one adaptation night followed by one experimental night of standard PSG at the laboratory, but only the experimental night was analyzed in the present study.

#### Dataset 2

This dataset included recordings from a total of 39 subjects: 23 young (aged 20–30 years, mean = 24.9 ± 0.5 years; 13 women, 10 men) and 16 elderly (aged 60–85 years, mean = 66.4 ± 0.8 years; 10 women, 6 men) adults. Data for both age groups were obtained from the publicly available “Bitbrain Open Access Sleep Dataset” PSG dataset hosted on OpenNeuro (47). PSG recordings acquired using a clinical Micromed system were used. This dataset includes subjects aged 18 to 82 years recruited from the general population with the goal of representing a broad range of characteristics and were screened to exclude severe medical, neurological, or psychiatric conditions. From this dataset, subjects were selected based on their age to form the two groups: we selected those aged 20–30 years for the young group and 60–85 years for the elderly group. Within this pool, we retained only the elderly participants who reached SWS during the night. Based on this subset, we then randomly selected a comparable number of young adults who also reached SWS and had good-quality EEG data, as determined by visual inspection.

#### Dataset 3

This dataset included recordings from a total of 40 subjects: 19 young (aged 25–35 years, mean = 28.7 ± 0.7 years; 10 women, 9 men) and 21 elderly (aged 65–85 years, mean = 69.8 ± 0.7 years; 10 women, 11 men) adults. Data for both age groups were obtained from the publicly available “Sleep-EDF Database Expanded” PSG dataset hosted on PhysioNet (48–50). Specifically, data from the Sleep Cassette Study was used, since this subset of data originates from a study on age-related effects on sleep in healthy adults (aged 25–101 years) who were not taking sleep-related medication. From this dataset, we selected those aged 25–35 years for the young group and 65–85 years for the elderly group. As in dataset 2, we retained only elderly participants who reached SWS. Only the experimental night was analyzed in the present study. As recordings were obtained using a wearable that continuously monitored the subjects at their homes for ∼20 h, recordings were analyzed starting from the “lights off” marks until the end of the night. This allowed us to exclude any potential daytime naps that were occasionally recorded following the main sleep period.

### Sleep data

All datasets included standard PSG recordings comprising electroencephalographic (EEG), electrooculographic, and chin electromyographic signals from full nights of sleep, along with their corresponding sleep stages manually scored by experts in 30-s epochs. However, as these datasets originated from independent studies, they slightly differ in acquisition systems and scoring protocols, which are detailed below.

#### Dataset 1

For both groups, EEG was recorded from six scalp electrodes (F3, F4, C3, C4, P3, and P4 according to the International 10-20 System) but referenced to the combination of both mastoids for young adults and to a single mastoid in elderly adults. Young adults' recordings were performed with BrainAmp amplifiers (Brain Products, Munich, Germany) at a sampling rate of 200 Hz, while elderly adults' recordings were performed with OpenBCI cyton amplifiers (OpenBCI, USA) at a sampling rate of 250 Hz. Sleep staging for both groups was based on the R&K scoring criteria (51). Since S3 and S4 were grouped together as SWS in the scoring of elderly adults, the same criteria were applied to the young adults' data. Certain recordings from the elderly group were fragmented into two files due to data acquisition interruptions caused by awakenings. The time elapsed between these files was accounted for in the calculation of total sleep time and awakenings.

#### Dataset 2

For both groups, EEG was recorded from six scalp electrodes (F3, F4, C3, C4, O1, and O2 according to the International 10-20 System), referenced to the left mastoid. Recordings were performed with a clinical Micromed system (Brain Quick Plus Evolution PSG system) at a sampling rate of 256 Hz. Sleep staging for both groups was based on the AASM scoring criteria (52).

#### Dataset 3

For both groups, EEG was recorded from four scalp electrodes (Fpz, Cz, Pz, and Oz according to the International 10-20 System), and two bipolar channels were obtained: Fpz-Cz and Pz-Oz. Recordings were performed with a wearable device consisting of a modified Walkman-like cassette-tape recorder (53) at a sampling rate of 100 Hz. Sleep staging for both groups was based on the R&K scoring criteria (51), but based on Fpz-Cz/Pz-Oz EEG channels instead of C4-A1/C3-A2 EEG channels, as suggested by van Sweden et al. (54). As in dataset 1, stages S3 and S4 were grouped together as SWS in both young and elderly adults.

#### Preprocessing and channel selection

To enable the inclusion of recordings from wearable devices with limited EEG channels, all analyses were performed on a single central EEG derivation per subject. Central derivations were chosen, as the probability of detecting an SO is the highest at central and frontal electrodes (2). For datasets 1 and 2, the best-quality central channel (C3 or C4) was selected for each subject based on visual inspection. For dataset 3, the Fpz-Cz derivation was used for all subjects. Provided sleep scorings were used, but additional unmarked artifacts were visually identified and excluded prior to analysis. Although the datasets differed in their sleep scoring criteria (using either R&K or AASM standards), we unified stage definitions to enable direct comparisons between age groups across all datasets. Specifically, stage 1 was defined as R&K S1 or AASM N1, stage 2 as R&K S2 or AASM N2, and SWS as R&K S3 + S4 or AASM N3. EEG signals were band-pass filtered between 0.3 and 35 Hz using a zero-phase finite impulse response (FIR) filter applied to the selected EEG channel. All preprocessing and analysis were conducted using Python (version 3.12.3), primarily with the MNE package (version 1.7.1) (55).

### SO detection

SOs (0.5–1 Hz) were automatically detected in artifact-free NREM (stage 2 and SWS) epochs using YASA slow-wave detection function (56), which is based on algorithms by Massimini et al. (2) and Carrier et al. (14). The frequency range parameter was set to YASA's default (0.3–1.5 Hz) due to the 0.2 Hz transition band of its FIR filter. The rest of the parameters used for SO detection were as follows: a negative deflection duration between 300 and 1,500 ms (default value), a positive deflection duration between 100 and 1,000 ms (default value), a negative peak amplitude below −40 µV with no upper limit, an unspecified positive peak amplitude, and a minimum PTP amplitude threshold of 75 µV for young adults (2) and 60 µV for elderly adults (15, 34) with no upper limit. This differential PTP amplitude criterion was set as a biological approach to account for the known reductions in SO amplitude associated with aging (12, 14, 15).

**Figure 5:**
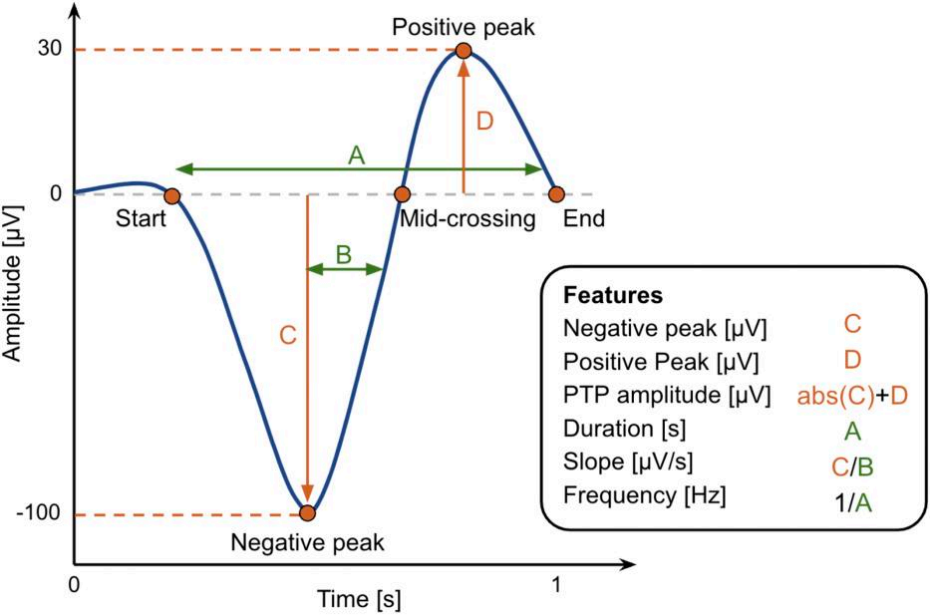
Schematic representation of the waveform features extracted from each detected SO. Feature A corresponds to the full duration of the wave (in seconds), encompassing both the negative and positive phases (i.e. the period). Feature B represents the duration (in seconds) of the rising phase between the negative peak and the following zero-crossing (mid-crossing). Feature C denotes the value of the negative peak (a negative value in µV), and D the value of the positive peak (in µV). Derived features include PTP amplitude, calculated as the sum of the absolute value of C and D (in µV); slope of the rising edge, calculated as the absolute value of the negative peak (in µV) divided by the rising edge duration B (in seconds); and frequency, defined as the inverse of the period, 1/A (in Hz).

SO features (duration, frequency, PTP amplitude, slope, and negative and positive peak amplitudes) were given by the YASA slow-wave detection algorithm (Fig. 5 shows a representation of how these features are calculated). For each subject, the mean of each of these features were calculated. Then the groups' mean features were calculated as the mean of the individual means. Proportions of stage 2 and SWS detections relative to the total number of NREM detections and detection densities (detections per unit of time) relative to the time spent on stage 2 and SWS were calculated.

### SO classification

Detected SOs were classified into two types based on the temporal proximity with their adjacent SOs: isolated SOs and consecutive SOs. We defined a temporal threshold (*δ*) to differentiate between these types. Isolated SOs were those occurring >*δ* seconds apart from both the preceding and subsequent SOs, while consecutive SOs were identified as SOs occurring <*δ* seconds apart from each other. Temporal distances (ISOIs) between successive SOs were measured between markers located at the negative peaks of each SO. *δ* was set to 2 s, based on the upper limit of the period of SOs. Given that SOs have a 0.5–1 Hz frequency range, their periods range between 1 and 2 s. Thus, if two SOs occur within 2 s of each other, they can be considered part of a sustained oscillatory pattern, like a continuous sinusoidal rhythm with a period of up to 2 s. Therefore, this separation allows us to distinguish between isolated events and more rhythmically organized patterns of SOs that we named as oscillatory trains. Oscillatory trains are defined as sequences of consecutive SOs, where the length of each train is determined by the number of consecutive SOs it comprises. The greater the number of consecutive SOs within a train, the longer its duration and the more sustained its rhythmic structure.

Proportions of each SO type (isolated and consecutive) relative to the total number of detected SOs were calculated to make results independent of the total amount of detections. The length of the oscillatory trains was calculated as the number of consecutive SOs that make up the train. Histograms were calculated for each subject (density histograms were chosen over frequency histograms to make results independent of the total amount of detections). Mean histograms were calculated for each group as the mean of individual histograms.

Additionally, we conducted a sensitivity analysis by progressively increasing the threshold value *δ*, starting from 0.5 s, and observing how the proportions of SO types changed. This analysis allowed us to ensure that the selected 2-s threshold provided a robust distinction between isolated and consecutive SOs.

These analyses were conducted across the entirety of NREM sleep (i.e. combining stage 2 and SWS), and additional analyses were performed to account for differences in SO density between age groups (14). Furthermore, stage-specific analyses were carried out separately for stage 2 and SWS to determine whether the observed group differences in SO dynamics are specific to a particular sleep stage or reflect more general alterations. This approach also allowed us to test whether our conclusions extend beyond the well-known reduction in SWS and relative increase in stage 2 commonly observed in elderly adults (12).

### SO density control analysis

To assess whether the observed differences in the temporal organization of SOs between age groups could be solely attributed to differences in SO density (14) or whether they reflect an age-related disruption in the temporal organization of SOs, we performed a control analysis in which SO density was kept constant within each sleep stage for each group while disrupting their observed temporal organization. To do this, the detected SO marker locations were randomized, ensuring that the total number of SOs per unit of time within each stage remained unchanged for each subject while eliminating their natural temporal structure. A minimum ISOIs of 0.5 s (the shortest ISOI observed in the empirical data; Fig. 2A) was ensured during the randomization. Then, the proportions of isolated and consecutive SOs, as well as the distribution of train lengths, were recalculated using the same methodology previously described. The randomization process was repeated 10 times, and the results were averaged. This analysis allowed us to determine whether the observed group differences were purely due to variations in SO density or whether they reflected fundamental changes in their temporal dynamics.

In addition, we conducted an epoch-based analysis to further investigate whether the temporal distribution of SOs (as isolated events or forming trains) differs between groups independently of differences in overall SO density. Instead of aggregating all detections across NREM sleep as a whole, we analyzed 30-s NREM epochs independently and calculated, for each epoch, the proportions of each SO type. This per-epoch proportion was then linked to the SO density of that same epoch, defined as the number of SOs detected within the epoch. Epochs were grouped according to their SO density for subjects within each group. This approach allowed us to compare the distribution of SO types across age groups for epochs with equivalent SO density, thus controlling for group differences in overall SO density. By comparing epochs with matched local density between age groups, we could determine whether SOs in elderly adults tend to appear more frequently as isolated events than in trains, even when the number of waves in a 30-s epoch is the same.

### Sleep structure and power analysis

The total sleep time (TST), time spent in each sleep stage (stage 1, stage 2, SWS, REM, and NREM [stage 2 + SWS]), and time spent awake after sleep onset (WASO) were calculated for all subjects. Sleep onset was defined as the first occurrence of a stage 2 epoch. The proportion of time spent in each stage relative to TST was determined, along with the percentages of stage 2 and SWS within total NREM sleep. SWS and REM sleep latencies from sleep onset were also measured. To further assess sleep fragmentation, in addition to WASO duration, the number of awakenings was quantified as the total count of transitions from NREM or REM sleep to wakefulness (W). Additionally, a SFI was computed as the total number of awakenings and shifts to stage 1 (from NREM or REM sleep) divided by TST in hours. Group comparisons were made for these measures to assess age-related differences in sleep architecture. Additionally, sleep fragmentation metrics (number of awakenings and SFI) were examined in relation to the proportion of isolated SOs to assess their potential contribution to age-related differences.

In addition, a PSD analysis was performed for each subject to assess the EEG power distribution across sleep stages and frequency rhythms of interest (SO: 0.5–1 Hz; delta: 1–4 Hz; theta: 4–8 Hz; alpha: 8–13 Hz; slow spindles: 9–12 Hz; fast spindles: 12–15 Hz; and beta: 15–30 Hz). For each sleep stage, the power of each rhythm was computed as the area under the curve of the corresponding PSD. The PSD was calculated using Welch's method, implemented through the psd_array_welch function from the mne.time_frequency module. Artifact-free epochs were segmented into 10-s consecutive blocks with a 50% overlap. Each block was tapered by a single Hanning window before applying Fast Fourier Transformation that resulted in block power spectra with a frequency resolution of 0.1 Hz. Power spectra were then averaged across all blocks. The power data were aggregated across subjects to calculate mean values for each group, allowing for comparative analysis.

### Statistics and reproducibility

All statistical analyses were conducted in R (version 4.4.1 (57)) and using SciPy (version 1.14.0 (58)) in Python. Plots were generated using Matplotlib (version 3.9.1 (59)). For the analysis of composite data, i.e. proportions that add up to one, we employed beta regression with random intercepts for data with two composites and Dirichlet regression for data with more than two composites. To implement the respective models, we used the R packages DirichletReg version 0.7.1 (60) for Dirichlet regression and glmmTMB version 1.1.10 (61) for beta regression. The groups were dummy coded with the “elderly” group as the reference category. The threshold for significance was set to *α* = 0.05.

## Supplementary Material

pgag108_Supplementary_Data

## Data Availability

All data used in this study are publicly available in their respective publications, except for the EEG data from the elderly adult group in dataset 1. These data were collected by the Laboratorio de Sueño y Memoria at the Instituto Tecnológico de Buenos Aires (ITBA) and are publicly available via the Open Science Framework (OSF) repository (https://osf.io/b6afs) (62).
