# Supplementary material for: Aging disrupts the temporal organization of slow oscillations beyond density reduction
Source: PNAS Nexus. 2026 Apr 16;5(4):pgag108. doi: 10.1093/pnasnexus/pgag108 (PMC13091574; doi:10.1093/pnasnexus/pgag108)
Supplement: pgag108_Supplementary_Data [file pgag108_supplementary_data.pdf]

Supplementary Information for

Aging disrupts the temporal organization of slow oscillations beyond density reduction

Lucila Capurro[1], Michael Radloff[2], María C. González[1], María L. Gorosito[1], Luis I. Brusco[3,4], Rodrigo Ramele[5], Cecilia Forcato[1*]
[1] Laboratorio de Sueño y Memoria, Departamento de Ciencias de la Vida, Instituto Tecnológico de Buenos Aires (ITBA), C1437 Ciudad Autónoma de Buenos Aires, Argentina.
[2] Department of Health Psychology, Institute for Psychology, University of Klagenfurt, 9020 Klagenfurt, Austria.
[3] Centro de Neuropsiquiatría y Neurología de la Conducta-CENECON, Facultad de Ciencias Médicas, Universidad de Buenos Aires (UBA), C1121A6B Ciudad Autónoma de Buenos Aires, Argentina.
[4] Consejo Nacional de Investigaciones Científicas y Técnicas (CONICET), C1425 Ciudad Autónoma de Buenos Aires, Argentina.
[5] Departamento de Ingeniería Informática, Instituto Tecnológico de Buenos Aires (ITBA), C1437 Ciudad Autónoma de Buenos Aires, Argentina.

*Cecilia Forcato, Iguazú 341, C1437 Ciudad Autónoma de Buenos Aires, Argentina
Email: cforcato@itba.edu.ar

This PDF file includes:

Supporting text
Figures S1 to S4
Tables S1 to S15

Supplementary Results

Age-related differences in the temporal organization of SOs

Stage-specific analyses of SO dynamics during NREM sleep (Stage 2 and SWS)

In both stage 2 and SWS, ISOI distributions (Fig. S2A,B) differed significantly between age groups (stage 2: two-sample Kolmogorov-Smirnov test: $D = 0.051$, $p < 0.001$; SWS: $D = 0.242$, $p < 0.001$).In both stages, distributions were concentrated below 2 s, with the highest-density bin at 1-1.5 s, supporting the $\delta = 2$ s threshold. Across both stages, young adults consistently showed a higher and narrower peak and a steeper decay, indicating more temporally clustered SOs. These differences were observed in both stages but were much stronger in SWS. In stage 2, age differences were modest (peak density: 0.236 vs. 0.176), and log-normal fits showed no significant difference in the shape parameter (σ young = 1.720, σ elderly = 1.725; $\Delta\sigma = -0.005$, 95% CI [-0.033, 0.021], $p = 0.672$). In SWS, differences were more pronounced: young adults showed a much higher peak (0.350 vs. 0.215) and a steeper decay, and elderly adults exhibited a significantly broader distribution (σ young = 1.140, σ elderly = 1.357; $\Delta\sigma = -0.217$, 95% CI [-0.254, -0.181], $p < 0.001$), consistent with a heavier-tailed ISOI distribution.

Elderly adults consistently displayed a higher proportion of isolated SOs across both stage 2 (Fig. S2C; mean ± SEM isolated proportion: young = 0.76 ± 0.01, elderly = 0.87 ± 0.02; Beta regression with random intercepts: $\beta 1 = 0.52$, $z = 4.24$, $p < 0.001$) and SWS (Fig. S2D; mean ± SEM isolated proportion: young = 0.47 ± 0.02, elderly = 0.70 ± 0.03; Beta regression with random intercepts: $\beta 1 = 0.65$, $z = 5.02$, $p < 0.001$). Furthermore, elderly adults exhibited shorter trains, with a higher proportion of trains of length 2 ($p < 0.001$) in both stage 2 and SWS, and a lower proportion of trains of length 3 ($p = 0.023$) in stage 2 and of trains of length 3-8 in SWS (all ps < 0.01), compared to younger adults (Fig. S2E,F). Full descriptive statistics (mean ± SEM) and complete Dirichlet regression results for each train length in each stage are provided in Tables S4,S5.

As in the global NREM analysis, varying δ supports that the observed group effects are robust and not contingent on the exact choice of $\delta = 2$ s (Fig. S2G,H).

Again, we analyzed the proportion of consecutive SOs as a function of SO density separately in stage 2 and SWS (Fig. S2I,J). In both stages, a positive association was observed, indicating that higher SO density was associated with a greater likelihood of SOs forming trains. Visual inspection revealed that younger adults consistently showed a higher proportion of consecutive SOs than elderly adults across comparable density levels. Multiple regression analysis with mean-centered SO density: (Stage 2: density = 0.11, $t(104) = 9.875$, $p < 0.001$; group = 0.08, $t(104) = 6.539$, $p < 0.001$; density x group = -0.00, $t(104) = -0.21$, $p = 0.828$; R-squared = 0.71; SWS: density = 0.038, $t(104) = 12.42$, $p < 0.001$; group = 0.10, $t(104) = 5.14$, $p < 0.001$; density x group = -0.01, $t(104) = -3.22$, $p = 0.002$; R-squared = 0.84). Multiple linear regression showed significant density x group interaction in SWS, but not in stage 2.

These results demonstrate that the age-related difference (higher proportion of isolated SOs and shorter trains in elderly adults) was present in both stages, indicating that the observed differences in NREM are not solely driven by the relative proportion of stage 2 and SWS within NREM sleep.

SO dynamics analyses across individual datasets (1, 2, and 3)

Across all three datasets, elderly adults consistently exhibited a higher proportion of isolated SOs than young adults (Fig. 4A-C). In dataset 1, the mean ± SEM was 0.77 ± 0.03 for elderly versus 0.52 ± 0.04 for young adults (Fig. 4A; Beta regression with random intercepts: $\beta 1 = 0.8454$, $z = 4.033$, $p < 0.001$). In dataset 2, elderly adults had 0.80 ± 0.04 compared to 0.57 ± 0.03 for young adults (Fig. 4B; Beta regression with random intercepts: $\beta 1 = 0.7645$, $z = 3.012$, $p = 0.003$), and in dataset 3, 0.87 ± 0.02 versus 0.59 ± 0.04 (Fig. 4C; Beta regression with random intercepts: $\beta 1 = 1.1633$, $z = 4.692$, $p < 0.001$).

The pattern for train lengths was also consistent across datasets (Fig. 4D-F). Elderly adults showed a higher proportion of the shortest possible trains (length = 2), which was significant in datasets 2 and 3 (dataset 2: $p = 0.003$; dataset 3: $p < 0.001$) but not in dataset 1 ($p = 0.592$). Conversely, young adults consistently exhibited higher proportions of longer trains (lengths 3-7 in datasets 1 and 2, 3-8 in

dataset 3; all ps < 0.05). Full descriptive statistics (mean ± SEM) and complete Dirichlet regression results for each train length in each dataset are provided in Tables S6-S8.

Varying the δ threshold revealed consistent patterns in all datasets (Fig. 4G-I). Young adults showed a steeper decline in the proportion of isolated SOs with increasing δ, and the smoothness of the curve supports that the age-related differences in SO temporal structure are not dependent on the exact choice of δ.

The relationship between global SO density and the proportion of consecutive SOs was preserved across datasets (Fig. 4J-L). In all datasets, a positive association between density and consecutive SO proportion was observed. Visual inspection revealed that younger adults consistently showed higher proportions of consecutive SOs than elderly adults at comparable density levels. Multiple regression with mean-centered SO density: (dataset 1: density = 0.65, $t(25) = 4.993$, $p < 0.001$; group = 0.12, $t(25) = 3.96$, $p = 0.008$; density x group = -0.007, $t(25) = -0.44$, $p = 0.663$; R-squared = 0.87; dataset 2: density = 0.094, $t(35) = 11.63$, $p < 0.001$; group = 0.11, $t(35) = 5.494$, $p < 0.001$; density x group = -0.023, $t(35) = -2.28$, $p = 0.029$; R-squared = 0.92; dataset 3: density = 0.08, $t(36) = 6.78$, $p < 0.001$; group = 0.18, $t(36) = 7.937$, $p < 0.001$; density x group = -0.03, $t(36) = -2.69$, $p = 0.011$; R-squared = 0.89). Multiple linear regression showed significant density x group interactions in dataset 2 and dataset 3, but not in dataset 1.

Finally, when analyses were restricted to epochs matched for SO density, the group differences persisted across all datasets (Fig. 4M-O). Young adults maintained higher proportions of consecutive SOs than elderly adults within intermediate density ranges (dataset 1: 3-6 SOs/epoch; dataset 2: 3-8 SOs/epoch; dataset 3: 2-7 SOs/epoch).

Standard sleep analyses (architecture, power, and SO features)

Sleep architecture

Elderly adults showed greater sleep fragmentation, with more awakenings, increased time spent awake after sleep onset (WASO), and more time spent in stage 1. Despite comparable TST, both NREM and REM sleep were markedly reduced, particularly SWS, which showed the most pronounced decline. Within NREM, elderly adults spent a higher proportion of time in stage 2 and a lower proportion in SWS, reflecting a redistribution toward lighter sleep. Full statistical details are reported in Table S9.

Power analysis

As expected, elderly adults showed a significant reduction in low-frequency activity (SO and delta bands) across all sleep stages, particularly during stage 2 and SWS, where fast spindle power was also significantly decreased. In addition, theta power was reduced in both SWS and REM sleep. Full statistical results are presented in Table S10.

SO quantity and features analysis

The number and density of detections were analyzed in NREM sleep as a whole and separately within stage 2 and SWS, allowing us to evaluate both the absolute contribution of each stage and their relative contribution, quantified as the proportion of SOs detected during NREM sleep that occurred in each stage. Elderly adults exhibited a lower number and density of SOs across NREM sleep compared to young adults, with the strongest reductions during SWS and a relative redistribution toward stage 2 (Table S11). In addition, elderly adults exhibited SOs with lower amplitudes, shallower slopes, attenuated peaks, slower frequency, and slightly longer durations (Table S12).

Morphological differences between isolated and consecutive SOs

Slow waves are not a homogeneous phenomenon and that multiple classes have been described in the literature, including distinctions between slow oscillations and K-complexes, as well as between different SO subtypes based on morphology and spatial extent (1-6). Therefore, we performed an exploratory analysis of waveform features comparing isolated and consecutive SOs within each age group (Fig. S4; Tables S13 and S14). This analysis shows that isolated and consecutive SOs differ in several morphological characteristics (e.g., amplitude, slope, and duration), indicating that our temporal classification captures physiologically meaningful differences in slow-wave activity.

Importantly, these differences were consistent across young and elderly adults. However, we believe that a direct comparison between our temporal categories and previously described slow-wave types, such as the type I and type II SOs reported by Bernardi et al. (2018) (2), would not be appropriate. Importantly, Bernardi et al. demonstrated that although type I SOs tend to be more temporally separated from each other (i.e., larger T1→T1 intervals), they frequently occur in close temporal proximity to type II SOs (T1→T2, T2→T1, or T2→T2), with inter-event intervals on the order of a few seconds (see Figure 6 from Bernardi et al., 2018: 10.3389/fnhum.2018.00248). As a result, both isolated and consecutive SO categories in our framework are likely to contain a mixture of these SO subtypes, preventing a one-to-one mapping between temporal organization and SO class.

References

1. Siclari, F., G. Bernardi, B. A. Riedner, J. J. LaRocque, R. M. Benca, G. Tononi, Two distinct synchronization processes in the transition to sleep: A high-density electroencephalographic study. Sleep 37, 1621-1637 (2014). https://doi.org/10.5665/sleep.4070
2. Bernardi, G., F. Siclari, G. Handjaras, B. A. Riedner, G. Tononi, Local and widespread slow waves in stable NREM sleep: Evidence for distinct regulation mechanisms. Front. Hum. Neurosci. 12, 248 (2018). https://doi.org/10.3389/fnhum.2018.00248
3. Malerba, P., L. N. Whitehurst, S. B. Simons, S. C. Mednick, Spatio-temporal structure of sleep slow oscillations on the electrode manifold and its relation to spindles. Sleep 42, zsy197 (2019). https://doi.org/10.1093/sleep/zsy197
4. Nghiem, T. E., N. Tort-Colet, T. Górski, U. Ferrari, S. Moghimyfiroozabad, J. S. Goldman, B. Teleńczuk, C. Capone, T. Bal, M. di Volo, A. Destexhe, Cholinergic switch between two types of slow waves in cerebral cortex. Cereb. Cortex 30, 3451-3466 (2020). https://doi.org/10.1093/cercor/bhz320
5. Bouchard, M., J. M. Lina, P. O. Gaudreault, A. Lafrenière, J. Dubé, N. Gosselin, J. Carrier, Sleeping at the switch. eLife 10, e64337 (2021). https://doi.org/10.7554/eLife.64337
6. Navarrete, M., A. Osorio-Forero, A. Gómez, D. Henao, F. E. Segura-Quijano, M. Le Van Quyen, M. Valderrama, Response of sleep slow oscillations to acoustic stimulation is evidenced by distinctive synchronization processes. Sleep 46, zsad110 (2023). https://doi.org/10.1093/sleep/zsad110

Figures and Tables

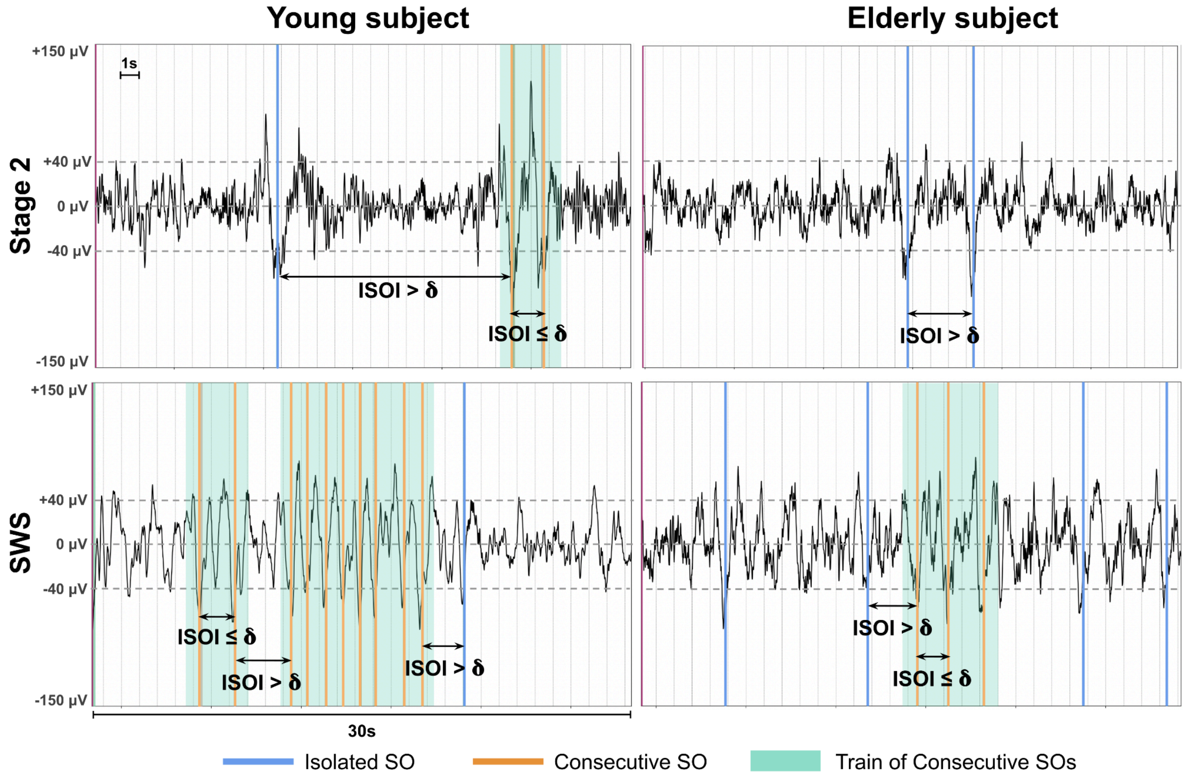

Figure S1. Examples of isolated and consecutive slow oscillations (SOs) during stage 2 and SWS epochs. Representative 30-s EEG segments from a young and an elderly subject (from dataset 1) illustrate the classification of detected SOs based on the inter-SO intervals (ISOIs) and a threshold δ. Blue lines indicate isolated SOs, orange lines indicate consecutive SOs, and green shading indicates trains of consecutive SOs. The selected central channel for each subject is shown, with signals preprocessed as described in the Materials and Methods section.

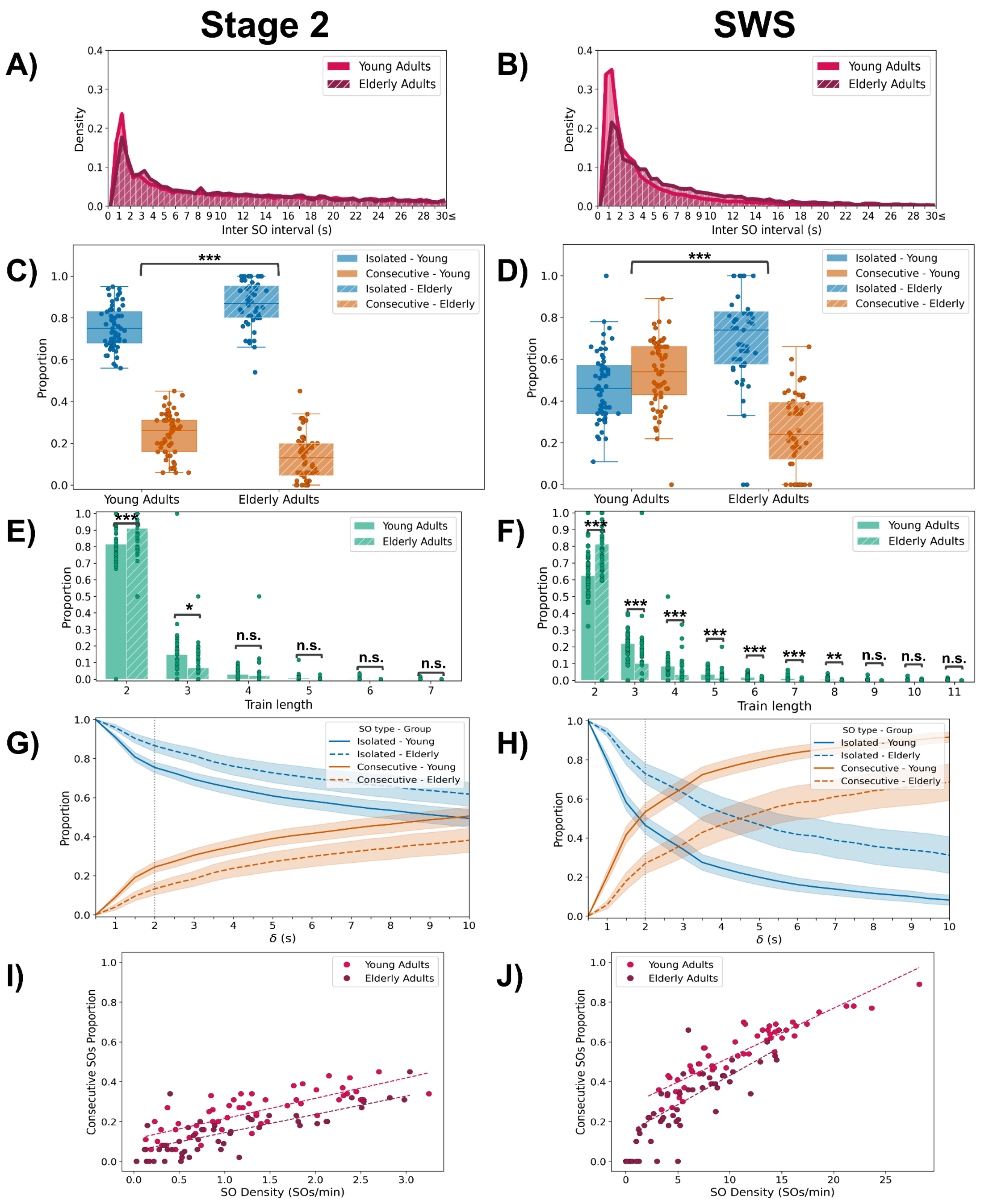

Figure S2. Stage-specific characterization of SO types and train structure during NREM sleep in the pooled dataset. Left column panels correspond to stage 2, while right column panels correspond to slow wave sleep (SWS). Data are shown for young (N = 57) and elderly (N = 51) adults. (A, B) Inter-SO interval density histograms showing the distribution of time (s) between successive SO negative peaks in young (solid magenta) and elderly (striped burgundy) adults. (C, D) Proportions of isolated (blue) and consecutive (orange) SOs across age groups. Boxplots show distributions for young (solid boxes) and elderly (striped boxes) adults. The statistical comparison tests whether the two-type composition of SOs differs between age groups. (E, F) Train length distributions. Density histograms indicate the relative frequency of trains of lengths 2 to 11, for young (solid bars) and elderly (striped bars) adults. (G, H) SO type proportions as a function of the inter-SO interval threshold (δ). Mean proportions of isolated (blue) and consecutive (orange) SOs are shown for δ ranging from

0.5 to 10 s. Solid lines indicate young adults; dashed lines indicate elderly adults. Shaded areas represent 95% confidence intervals. The vertical dotted line marks the 2-s threshold used in the main analysis. (I, J) Relationship between global SO density (waves/minute of stage 2 or SWS) and the proportion of consecutive SOs per subject. Linear fits are shown for young (magenta) and elderly (burgundy) adults. ***$p < 0.001$; **$p < 0.01$; *$p < 0.05$.

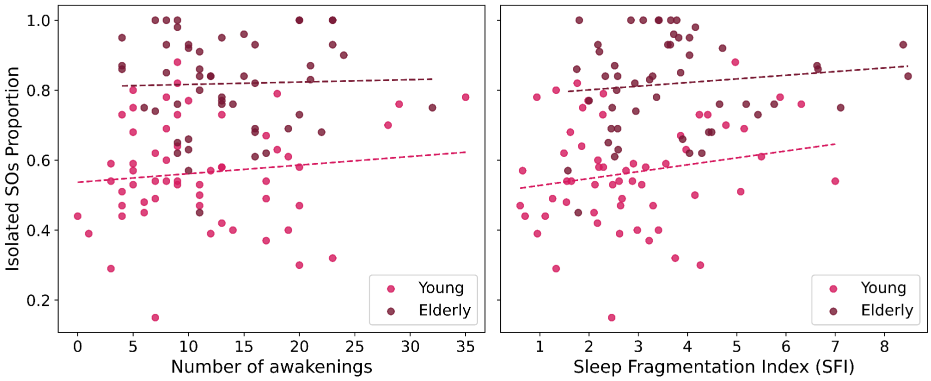

Figure S3. Relationship between sleep fragmentation and the proportion of isolated SOs per subject. Left panel: proportion of isolated SOs as a function of the number of awakenings. Right panel: proportion of isolated SOs as a function of the sleep fragmentation index (SFI). Data are shown for young (N = 57) and elderly (N = 51) adults. Linear fits are shown for young (magenta) and elderly (burgundy) adults.

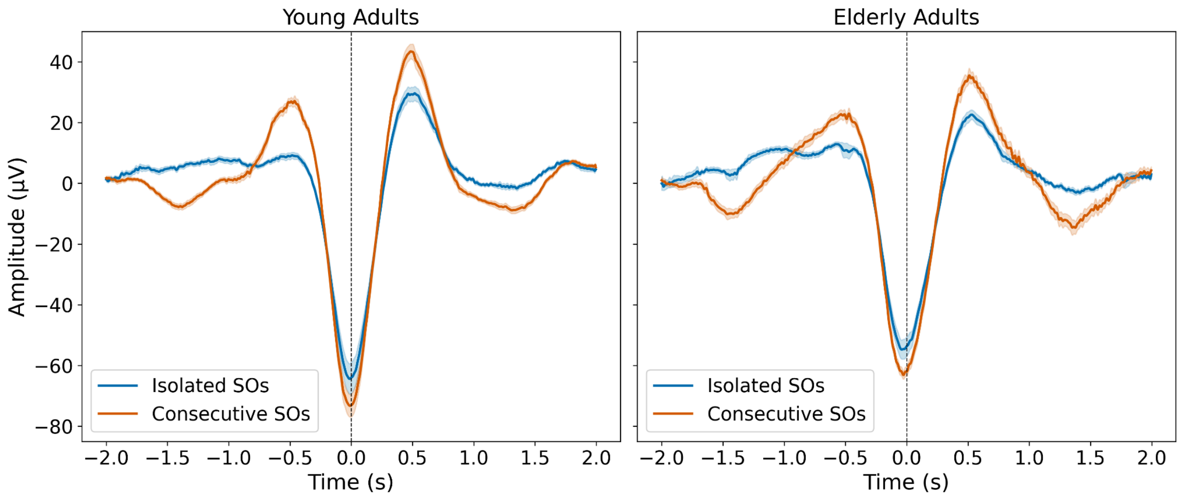

Figure S4. Grand-average SO waveforms for isolated (blue) and consecutive (orange) SOs are shown for young (left) and elderly (right) adults. Shaded regions represent SEM across participants. Waveforms are time-locked to the negative peak (0 s, dashed line).

Table S1. Results of Dirichlet regression comparing train length proportions between young and elderly adults during NREM sleep in the pooled dataset.

Train length	Young adults	Elderly adults	β1	z	p
2	0.671 ± 0.014	0.861 ± 0.023	1.069	7.99	**< 0.001 *****
3	0.197 ± 0.007	0.110 ± 0.022	2.462	15.69	**< 0.001 *****
4	0.072 ± 0.006	0.022 ± 0.005	1.826	10.84	**< 0.001 *****
5	0.030 ± 0.003	0.005 ± 0.001	1.285	7.23	**< 0.001 *****
6	0.014 ± 0.002	0.001 ± 0.000	0.908	4.96	**< 0.001 *****
7	0.008 ± 0.001	0.000 ± 0.000	0.757	4.10	**0.035 ***
8	0.004 ± 0.001	0.000 ± 0.000	0.604	3.25	**0.001 ****
9	0.002 ± 0.001	0.000 ± 0.000	0.430	2.30	**0.021 ***
10	0.001 ± 0.000	0.000 ± 0.000	0.372	1.99	**0.047 ***
11	0.001 ± 0.000	0.000 ± 0.000	0.375	2.00	**0.045 ***

*Mean ± SEM values are shown for each group. Dirichlet regression with the "elderly" group being the reference category was used. β1 reflects the group difference. Significant p-values are highlighted in bold. ***, p < 0.001; **, p < 0.01; *, p < 0.05.*

Table S2. Results of Dirichlet regression testing the interaction between age group (young vs. elderly) and dataset type (observed vs. randomized) on train length proportions during NREM sleep in the pooled dataset.

Train length	Young adults	Elderly adults	β3	z	p
2	0.899 ± 0.010	0.947 ± 0.007	-1.173	-6.18	**< 0.001** ***
3	0.085 ± 0.007	0.047 ± 0.006	-1.346	-6.05	**< 0.001** ***
4	0.013 ± 0.002	0.005 ± 0.001	-1.282	-5.18	**< 0.001** ***
5	0.002 ± 0.001	0.000 ± 0.000	-1.053	-4.07	**< 0.001** ***
6	0.001 ± 0.000	0.000 ± 0.000	-0.789	-3.00	**0.003** **
7	0.000 ± 0.000	0.000 ± 0.000	-0.716	-2.71	**0.007** **
8	0.000 ± 0.000	0.000 ± 0.000	-0.575	-2.17	**0.030** *
9	0.000 ± 0.000	0.000 ± 0.000	-0.391	-1.47	0.141
10	0.000 ± 0.000	0.000 ± 0.000	-0.354	-1.33	0.182
11	0.000 ± 0.000	0.000 ± 0.000	-0.343	-1.29	0.200

*Mean ± SEM values are shown for each group. Dirichlet regression with the "elderly" group and "observed" dataset being the reference categories, respectively, was used. β3 reflects the interaction between group and dataset (observed vs. randomized). Significant p-values are highlighted in bold. ***, p < 0.001; **, p < 0.01; *, p < 0.05.*

Table S3. Results of Dirichlet regression comparing train length proportions between young and elderly adults during NREM sleep in the randomized pooled dataset.

Train length	Young adults	Elderly adults	β1	z	p
2	0.899 ± 0.010	0.947 ± 0.007	-0.100	-0.80	0.423
3	0.085 ± 0.007	0.047 ± 0.006	1.004	6.78	**< 0.001 *****
4	0.013 ± 0.002	0.005 ± 0.001	0.453	2.62	**0.009 ****
5	0.002 ± 0.001	0.000 ± 0.000	0.162	0.89	0.371
6	0.001 ± 0.000	0.000 ± 0.000	0.059	0.32	0.747
7	0.000 ± 0.000	0.000 ± 0.000	0.003	0.02	0.986
8	0.000 ± 0.000	0.000 ± 0.000	-0.009	-0.05	0.960
9	0.000 ± 0.000	0.000 ± 0.000	-0.009	-0.05	0.960
10	0.000 ± 0.000	0.000 ± 0.000	-0.031	-0.17	0.865

*Mean ± SEM values are shown for each group. Dirichlet regression with the "elderly" group being the reference category was used. β1 reflects the group difference. Significant p-values are highlighted in bold. ***, p < 0.001; **, p < 0.01; *, p < 0.05.*

Table S4. Results of Dirichlet regression comparing train length proportions between young and elderly adults during S2 sleep in the pooled dataset.

Train length	Young adults	Elderly adults	β1	z	p
2	0.816 ± 0.020	0.910 ± 0.017	-0.944	-5.36	**< 0.001 *** **
3	0.147 ± 0.019	0.067 ± 0.013	0.420	2.27	**0.023 * **
4	0.028 ± 0.004	0.021 ± 0.010	0.115	0.61	0.542
5	0.006 ± 0.002	0.001 ± 0.001	-0.066	-0.35	0.727
6	0.002 ± 0.001	0.000 ± 0.000	-0.133	-0.70	0.484
7	0.001 ± 0.000	0.001 ± 0.001	-0.177	-0.93	0.351

Mean ± SEM values are shown for each group. Dirichlet Regression with the "elderly" group being the reference category was used. β1 reflects the group difference. Significant p-values are highlighted in bold.

Table S5. Results of Dirichlet regression comparing train length proportions between young and elderly adults during SWS sleep in the pooled dataset.

Train length	Young adults	Elderly adults	β1	z	p
2	0.626 ± 0.016	0.812 ± 0.035	0.713	5.08	**< 0.001** ***
3	0.219 ± 0.010	0.101 ± 0.023	2.319	14.07	**< 0.001** ***
4	0.084 ± 0.009	0.033 ± 0.009	1.480	8.43	**< 0.001** ***
5	0.036 ± 0.004	0.012 ± 0.004	1.023	5.59	**< 0.001** ***
6	0.017 ± 0.002	0.002 ± 0.001	0.742	3.97	**< 0.001** ***
7	0.009 ± 0.002	0.000 ± 0.000	0.623	3.31	**< 0.001** ***
8	0.005 ± 0.001	0.000 ± 0.000	0.504	2.67	**0.008** **
9	0.002 ± 0.001	0.000 ± 0.000	0.332	1.75	0.081
10	0.001 ± 0.001	0.000 ± 0.000	0.270	1.42	0.156
11	0.001 ± 0.000	0.000 ± 0.000	0.280	1.47	0.141

*Mean ± SEM values are shown for each group. Dirichlet regression with the "elderly" group being the reference category was used. β1 reflects the group difference. Significant p-values are highlighted in bold. ***, p < 0.001; **, p < 0.01; *, p < 0.05.*

Table S6. Results of Dirichlet regression comparing train length proportions between young and elderly adults during NREM sleep in dataset 1.

Train length	Young adults	Elderly adults	β1	z	p
2	0.641 ± 0.027	0.877 ± 0.027	0.112	0.54	0.592
3	0.210 ± 0.010	0.101 ± 0.023	1.757	7.26	**< 0.001** ***
4	0.079 ± 0.010	0.014 ± 0.004	1.750	6.21	**< 0.001** ***
5	0.034 ± 0.007	0.007 ± 0.003	1.088	3.59	**< 0.001** ***
6	0.020 ± 0.004	0.000 ± 0.000	1.132	3.60	**< 0.001** ***
7	0.008 ± 0.002	0.000 ± 0.000	0.688	2.11	**0.035** *
8	0.005 ± 0.001	0.000 ± 0.000	0.531	1.61	0.108
9	0.002 ± 0.001	0.000 ± 0.000	0.370	1.11	0.269
10	0.000 ± 0.000	0.001 ± 0.001	0.205	0.61	0.543
11	0.001 ± 0.000	0.000 ± 0.000	0.259	0.77	0.442

*Mean ± SEM values are shown for each group. Dirichlet regression with the "elderly" group being the reference category was used. β1 reflects the group difference. Significant p-values are highlighted in bold. ***, p < 0.001; **, p < 0.01; *, p < 0.05.*

Table S7. Results of Dirichlet regression comparing train length proportions between young and elderly adults during NREM sleep in dataset 2.

Train length	Young adults	Elderly adults	β1	z	p
2	0.685 ± 0.021	0.854 ± 0.032	0.576	2.96	**0.003** **
3	0.194 ± 0.010	0.111 ± 0.025	2.144	9.03	**< 0.001** ***
4	0.062 ± 0.007	0.026 ± 0.008	1.496	5.59	**< 0.001** ***
5	0.033 ± 0.005	0.006 ± 0.002	1.396	4.98	**< 0.001** ***
6	0.014 ± 0.003	0.002 ± 0.001	0.904	3.09	**0.002** **
7	0.007 ± 0.002	0.001 ± 0.001	0.706	2.37	**0.018** *
8	0.003 ± 0.001	0.000 ± 0.000	0.512	1.70	0.090
9	0.001 ± 0.001	0.000 ± 0.000	0.385	1.27	0.206
10	0.001 ± 0.000	0.000 ± 0.000	0.385	1.27	0.206
11	0.001 ± 0.000	0.000 ± 0.000	0.373	1.23	0.221

*Mean ± SEM values are shown for each group. Dirichlet regression with the "elderly" group being the reference category was used. β1 reflects the group difference. Significant p-values are highlighted in bold. ***, p < 0.001; **, p < 0.01; *, p < 0.05.*

Table S8. Results of Dirichlet regression comparing train length proportions between young and elderly adults during NREM sleep in dataset 3.

Train length	Young adults	Elderly adults	β1	z	p
2	0.679 ± 0.026	0.857 ± 0.048	1.436	6.77	**< 0.001** ***
3	0.191 ± 0.016	0.115 ± 0.048	2.436	9.66	**< 0.001** ***
4	0.081 ± 0.015	0.024 ± 0.009	1.993	7.49	**< 0.001** ***
5	0.023 ± 0.005	0.003 ± 0.002	1.213	4.22	**< 0.001** ***
6	0.009 ± 0.003	0.001 ± 0.000	0.759	2.55	**0.011** *
7	0.008 ± 0.003	0.000 ± 0.000	0.799	2.68	**0.007** **
8	0.006 ± 0.002	0.000 ± 0.000	0.724	2.42	**0.016** *
9	0.002 ± 0.002	0.000 ± 0.000	0.551	1.83	0.068
10	0.001 ± 0.001	0.000 ± 0.000	0.516	1.71	0.088
11	0.001 ± 0.001	0.000 ± 0.000	0.495	1.64	0.102

*Mean ± SEM values are shown for each group. Dirichlet regression with the "elderly" group being the reference category was used. β1 reflects the group difference. Significant p-values are highlighted in bold. ***, p < 0.001; **, p < 0.01; *, p < 0.05.*

Table S9. Sleep architecture parameters compared between young and elderly adults.

	Young adults	Elderly adults	Statistic, p-value
TST (min)	441.9 ± 7.5	411.7 ± 10.3	W = -1.8, p = 0.072
Stage 1 (min)	22.3 ± 2.4	41.0 ± 4.4	**W = 4.0, p < 0.001 *** **
Stage 2 (min)	249.6 ± 7.7	216.8 ± 7.8	**W = -2.5, p = 0.011 * **
SWS (min)	55.7 ± 4.4	29.4 ± 4.5	**W = -4.2, p < 0.001 *** **
REM (min)	83.1 ± 4.0	68.4 ± 3.8	**W = -2.5, p = 0.014 * **
NREM (min)	305.3 ± 6.6	246.2 ± 7.7	**W = -4.9, p < 0.001 *** **
NREM + REM (min)	388.4 ± 8.0	314.7 ± 9.8	**W = -5.0, p < 0.001 *** **
WASO (min)	22.6 ± 3.3	54.8 ± 5.6	**W = 5.1, p < 0.001 *** **
Stage 1 (%)	5.0 ± 0.5	9.9 ± 0.9	β1 = -0.262, z = -1.56, p = 0.119
Stage 2 (%)	56.5 ± 1.4	53.3 ± 1.6	**β1 = 0.376, z = 2.503, p = 0.012 * **
SWS (%)	12.7 ± 1.0	7.1 ± 1.0	**β1 = 1.003, z = 5.97, p < 0.001 *** **
REM (%)	18.7 ± 0.8	16.4 ± 0.8	**β1 = 0.404, z = 2.56, p = 0.0104 * **
WASO (%)	5.1 ± 0.7	13.0 ± 1.3	**β1 = -0.592, z = -3.53, p < 0.001 *** **
Stage 2/NREM (%)	81.4 ± 1.4	88.2 ± 1.7	**β1 = -0.079, z = -2.90, p = 0.004 ** **
SWS/NREM (%)	18.6 ± 1.4	11.8 ± 1.7	**(Complement of Stage 2/NREM)**
SWS latency (min)	39.8 ± 7.7	44.5 ± 6.2	W = 1.0, p = 0.313
REM latency (min)	108.7 ± 8.1	94.9 ± 8.7	W = -1.5, p = 0.132
Awakenings	10.9 ± 1.0	13.5 ± 0.8	**W = 2.5, p = 0.011 * **
SFI	2.8 ± 0.2	3.7 ± 0.2	**W = 3.1, p = 0.002 ** **

*Mean ± SEM values are shown for each group. Wilcoxon rank-sum tests were used for group comparisons of continuous variables, whereas Beta regression with random intercepts and Dirichlet regression (with the "elderly" group as reference) were applied to proportions with two and more than two composites, respectively. For differences in proportions, β1 reflects the group difference. Significant p-values are highlighted in bold. ***, $p < 0.001$; **, $p < 0.01$; *, $p < 0.05$.*

Table S10. Absolute EEG power in different frequency bands across sleep stages in young and elderly adults.

Sleep stage		Frequency band	Mean Absolute Power (μV²)		Statistic, p-value
			Young adults	Elderly adults	
NREM	Stage 2	SO	1968 ± 258	1058 ± 109	**W = -3.6, p < 0.001 *** **
		Delta	2075 ± 238	1124 ± 73	**W = -4.0, p < 0.001 *** **
		Theta	301 ± 25	255 ± 21	W = -1.2, p = 0.213
		Slow spindles	87 ± 9	92 ± 8	W = 1, p = 0.326
		Fast spindles	81 ± 6	53 ± 4	**W = -3.2, p = 0.002 ** **
		Alpha	151 ± 15	156 ± 13	W = 0.6, p = 0,528
		Beta	70 ± 8	85 ± 11	W = 1.3, p = 0.185
	SWS	SO	3582 ± 466	1311 ± 193	**W = -3.2, p = 0.002 ** **
		Delta	3416 ± 445	1282 ± 143	**W = -3.5, p < 0.001 *** **
		Theta	354 ± 33	275 ± 31	**W = -2.0, p = 0.047 * **
		Slow spindles	75 ± 7	91 ± 11	W = 0.9, p = 0.377
		Fast spindles	75 ± 8	46 ± 4	**W = -2.7, p = 0.007 ** **
		Alpha	136 ± 12	150 ± 16	W = 0.3, p = 0.747
		Beta	63 ± 19	53 ± 5	W = 1.9, p = 0.052
REM		SO	1209 ± 188	662 ± 88	**W = -2.3, p = 0.020 * **
		Delta	1446 ± 173	840 ± 79	**W = -2.6, p = 0.011 * **
		Theta	261 ± 19	209 ± 20	**W = -2.3, p = 0.022 * **
		Slow spindles	72 ± 6	74 ± 7	W = 0.5, p = 0.616

	Fast spindles	63 ± 7	42 ± 4	W = -1.9, p = 0.062
	Alpha	126 ± 11	129 ± 11	W = 0.2, p = 0.808
	Beta	65 ± 10	86 ± 11	W = 1.7, p = 0.087

*Mean absolute EEG power (μV²) ± SEM are shown for each group. Wilcoxon rank-sum tests were used to compare groups. Significant p-values are highlighted in bold. ***, p < 0.001; **, p < 0.01; *, p < 0.05.*

Table S11. Slow oscillation counts, stage-specific proportions, and densities in stage 2, SWS, and total NREM sleep for young and elderly adults.

	Young adults	Elderly adults	Statistic, p-value
Stage 2	325.0 ± 27.9	213.0 ± 29.0	**W = -3.2, p = 0.001 ****
SWS	684.5 ± 91.8	190.1 ± 39.4	**W = -5.4, p < 0.001 *****
NREM	1009.5 ± 101.5	403.2 ± 58.4	**W = -5.2, p < 0.001 *****
Stage 2/NREM	0.4 ± 0.0	0.7 ± 0.0	**β1 = -1.097, z = -5.50, p < 0.001 *****
SWS/NREM	0.6 ± 0.0	0.3 ± 0.0	**(Complement of Stage 2/NREM)**
δStage 2 (n/min)	1.3 ± 0.1	1.0 ± 0.1	**W = -2.1, p = 0.038 ***
δSWS (n/min)	10.7 ± 0.8	5.4 ± 0.6	**W = -4.9, p < 0.001 *****
δNREM (n/min)	3.3 ± 0.3	1.6 ± 0.2	**W = -4.3, p < 0.001 *****

*Mean number of detections in stage 2, SWS, and total NREM (stage 2 + SWS), mean proportions of stage 2 and SWS detections relative to total NREM detections, and mean SO densities (δ, detections per minute) in stage 2, SWS, and NREM, shown as mean ± SEM for each group. Wilcoxon rank-sum tests were used for group comparison on continuous data, while Beta regression with random intercepts was used for proportions. For differences in proportions, β1 reflects the group difference. Significant p-values are highlighted in bold. ***, p < 0.001; **, p < 0.01; *, p < 0.05.*

Table S12. Slow oscillation features in young and elderly adults.

	Young adults	Elderly adults	Statistic, p-value
Count	1009.5 ± 101.5	403.2 ± 58.4	**W = -5.2, p < 0.001 *** **
PTP amplitude (μV)	118.8 ± 2.3	90.9 ± 1.6	**W = -7.5, p < 0.001 *** **
Negative peak (μV)	-66.6 ± 1.3	-54.4 ± 0.7	**W = 6.5, p < 0.001 *** **
Positive peak (μV)	52.2 ± 1.1	36.6 ± 0.9	**W = -7.9, p < 0.001 *** **
Slope (μV/s)	446.7 ± 9.2	292.6 ± 7.2	**W = -8.6, p < 0.001 *** **
Duration (s)	1.2 ± 0.0	1.3 ± 0.0	**W = 5.4, p < 0.001 *** **
Frequency (Hz)	0.9 ± 0.0	0.8 ± 0.0	**W = -5.1, p < 0.001 *** **

*Mean ± SEM values are shown for each group. Wilcoxon rank-sum tests were used. Significant p-values are highlighted in bold. ***, p < 0.001; **, p < 0.01; *, p < 0.05.*

Table S13. Comparison of slow oscillation features between isolated and consecutive SOs in young adults.

	Isolated SOs	Consecutive SOs	Mean difference	Statistic, p-value
PTP amplitude (μV)	113.1 ± 1.9	125.6 ± 2.7	12.5 ± 1.6	**W = 1, p < 0.001**
Negative peak (μV)	-65.6 ± 1.2	-67.8 ± 1.4	-2.2 ± 0.7	**W = 393, p = 0.001**
Positive peak (μV)	47.5 ± 0.7	57.8 ± 1.3	10.3 ± 1.0	**W = 0, p < 0.001**
Slope (μV/s)	407.3 ± 6.6	493.1 ± 10.0	85.8 ± 6.9	**W = 0, p < 0.001**
Duration (s)	1.3 ± 0.0	1.2 ± 0.0	-0.1 ± 0.0	**W = 2.5, p < 0.001**
Frequency (Hz)	0.8 ± 0.0	0.9 ± 0.0	0.1 ± 0.0	**W = 2.5, p < 0.001**

*Mean ± SEM values are shown for each SO type. Wilcoxon signed-rank tests were used. Significant p-values are highlighted in bold. ***, p < 0.001; **, p < 0.01; *, p < 0.05.*

Table S14. Comparison of slow oscillation features between isolated and consecutive SOs in elderly adults.

	Isolated SOs	Consecutive SOs	Mean difference	Statistic, p-value
PTP amplitude (μV)	90.3 ± 1.3	103.5 ± 2.6	14.9 ± 2.5	**W = 17, p < 0.001**
Negative peak (μV)	-54.8 ± 0.6	-56.7 ± 1.2	-2.1 ± 1.2	W = 372, p = 0.154
Positive peak (μV)	35.5 ± 0.6	46.8 ± 1.6	12.8 ± 1.7	**W = 11, p < 0.001**
Slope (μV/s)	287.7 ± 6.3	357.3 ± 11.9	84.6 ± 1.3	**W = 34, p < 0.001**
Duration (s)	1.3 ± 0.0	1.3 ± 0.0	-0.1 ± 0.0	**W = 192, p = 0.001**
Frequency (Hz)	0.8 ± 0.0	0.8 ± 0.0	0.1 ± 0.0	**W = 172, p < 0.001**

*Mean ± SEM values are shown for each SO type. Wilcoxon signed-rank tests were used. Significant p-values are highlighted in bold. ***, p < 0.001; **, p < 0.01; *, p < 0.05.*

Supplementary Materials and Methods

Subjects

Table S15. Subject codes included in the analyses for each publicly available dataset.

Dataset	Group	Subjects codes
Dataset 1	Young adults	S61, S62, S63, S64, S65, S66, S67, S68, S69, S70, S71, S72, S73, S74, S75
Dataset 2	Young adults	sub-1, sub-2, sub-3, sub-5, sub-11, sub-14, sub-25, sub-26, sub-28, sub-29, sub-44, sub-46, sub-48, sub-49, sub-104, sub-105, sub-106, sub-108, sub-122, sub-123, sub-126, sub-127, sub-128
	Elderly adults	sub-17, sub-19, sub-21, sub-32, sub-37, sub-39, sub-40, sub-41, sub-50, sub-54, sub-91, sub-109, sub-110, sub-111, sub-120, sub-121
Dataset 3	Young adults	SC400, SC401, SC402, SC403, SC404, SC405, SC406, SC407, SC408, SC409, SC410, SC411, SC412, SC414, SC415, SC416, SC417, SC418, SC419
	Elderly adults	SC440, SC441, SC442, SC443, SC444, SC445, SC446, SC447, SC448, SC449, SC450, SC451, SC452, SC453, SC454, SC455, SC456, SC457, SC458, SC459, SC477

Subject codes correspond to identifiers in the original datasets.
